# Selective Role of Mevalonate Pathway in Regulating Perforin but Not FasL and TNFalpha Release in Human Natural Killer Cells

**DOI:** 10.1371/journal.pone.0062932

**Published:** 2013-05-07

**Authors:** Alessandro Poggi, Silvia Boero, Alessandra Musso, Maria Raffaella Zocchi

**Affiliations:** 1 Molecular Oncology and Angiogenesis Unit, IRCCS AOU San Martino – IST National Institute for Cancer Research, Genoa, Italy; 2 Division of Immunology, Transplants and Infectious Diseases, Scientific Institute San Raffaele, Milan, Italy; INSERM-Université Paris-Sud, France

## Abstract

We have analyzed the effects of fluvastatin, an inhibitor of the enzyme 3-hydroxy-3-methylglutaryl-coenzyme A (HMG-CoA) reductase involved in mevalonate synthesis, on human NK cell-mediated anti-tumor cytolysis. Fluvastatin inhibited the activation of the small guanosin triphosphate binding protein (GTP) RhoA and the consequent actin redistribution induced by ligation of LFA1 involved in NK-tumor target cell adhesion. Also, fluvastatin reduced ganglioside M1 rafts formation triggered through the engagement of NK cell activating receptors as FcγRIIIA (CD16), NKG2D and DNAM1. Cytolysis of tumor targets was inhibited up to 90% when NK cells were cultured with fluvastatin by affecting i) receptor-mediated increase of the intracellular free calcium concentration, ii) activation of akt1/PKB and iii) perforin and granzyme release. Fluvastatin displayed a stronger inhibiting effect on NKG2D, DNAM1, 2B4, NKp30, NKp44 and NKp46 than on CD16-mediated NK cell triggering. This was in line with the impairment of surface expression of all these receptors but not of CD16. Remarkably, fluvastatin did not affect the expression of the inhibiting receptors CD94, KIR2D and LAIR1. FasL release elicited by either NK-tumor cell interaction or CD16 or NKG2D engagement, as well as FasL-mediated killing, were not sensitive to fluvastatin. Moreover, TNFα secretion triggered in NK cells upon incubation with tumor target cells or engagement of NKG2D receptor was not impaired in fluvastatin-treated NK cells. Likewise, antibody dependent cellular cytotoxicity (ADCC) triggered through FcγRIIIA engagement with the humanized monoclonal antibody rituximab or trastuzumab was only marginally affected in fluvastatin-treated NK cells. Altogether these findings suggest that interference with mevalonate synthesis impairs activation and assembly of cytoskeleton, degranulation and cytotoxic effect of perforins and granzyme but not FasL- and TNFα-mediated cytotoxicity.

## Introduction

Natural Killer (NK) cells can exert their anti-tumor effector function through degranulation and release of perforins and granzymes [1]–[5]. The interaction between LFA1 on NK cells and ICAM1 on target cells plays a key role both in the adhesive phase and in the activation of NK cells [5], [6].

Several activating receptors as NKG2D, DNAM1, natural cytotoxicity receptors (NCR) and 2B4 are involved as well, depending on whether the corresponding ligands are expressed on tumor target cells [6]–[11]. In addition, the employment in cancer therapy of human antibodies to either CD20 or HER2^+^ have shown to improve the outcome of CD20^+^ B lymphoproliferative diseases as non-Hodgkin lymphomas (NHL) and chronic lymphocytic leukemia (CLL) or HER2^+^ breast adenocarcinomas respectively [12], [13]. The therapeutic role of these antibodies is thought to be, at least in part, dependent on the antibody dependent cellular cytotoxicity (ADCC) triggered through the engagement of FCγRIIIA (CD16) expressed on NK cells [1], [9], [10]. Upon ligation of activating receptors, NK cells can also release cytotoxic molecules as FasL and/or TNFα which kill tumor target cells [1]–[5]. Perforin-mediated killing is mainly calcium dependent and it is effective after NK-target cell interaction within a few hours (1–3 h) while FasL- and TNFα-mediated cytotoxicity is mostly calcium independent and it is evident after a longer time (18–48 h) [1]. Indeed, FasL or TNFα should first interact with their specific receptors which in turn lead to programmed cell death of tumor targets [1]. Both cytolytic mechanisms contribute to eliminate tumor cells; thus, anti-tumor drugs employed to limit cell proliferation and survival should not interfere with these mechanisms of killing, in order to maintain the anti-tumor immune response. Statins, inhibitors of the 3-hydroxy-3-methylglutaryl coenzyme A (HMG-CoA) reductase and potent blockers of the synthesis of mevalonate, the precursor of cholesterol [14], [15], have been proposed as anti-neoplastic drugs [17]–[21]. Indeed, as cholesterol is a key component of eukaryotic cell membranes [16] the effect of statins may lead to the inhibition of tumor cell proliferation and survival [17]–[21]. However, statins can affect also the function of immune-competent cells and this effect may be the major drawback for the employment of these drugs in anti-tumor treatment [22]–[24]. Previous reports showed that lipophilic statins inhibit NK cell-mediated cytolysis of tumor targets by interfering with the secretion of perforins [25]. This interference is associated with a reduction of the polarization of cytolytic granules that follows the formation of LFA1-mediated effector-target cell conjugates [26]. However, it is not known whether in NK cells statins can affect the LFA1-mediated activation of the small guanosin triphosphate (GTP) binding protein RhoA, which play a key role in cell adhesion and motility [27]. It has been also shown that transcription of FasL mRNA is not inhibited by statins in NK cells [25], although it remains to be determined whether or not FasL secretion and FasL-mediated cytolysis are affected. Furthermore, it is of interest to elucidate whether the secretion of TNFα elicited from NK cells upon interaction with tumor targets is still effective in the presence of statins. Recently, it has been reported that IL-2 co-stimulation enables statin-mediated IFNγ production by human peripheral blood NK cells in the presence of CD14^+^CD56^+^ dendritic cells [28], suggesting that statins can also trigger, beside inhibit, NK cells.

Herein, we show that fluvastatin does not affect the release of FasL and TNFα and the consequent killing of tumor target cells due to these soluble factors. Also, ADCC triggered by the humanized monoclonal antibody rituximab or trastuzumab was only marginally affected in fluvastatin-treated NK cells On the other hand, fluvastatin can impair: a) LFA1-mediated activation of RhoA and the consequent redistribution of actin filaments conceivably involved in the release of cytotoxic granules; b) membrane raft arrangement and cytolysis of targets occurring upon engagement of activating receptors; c) intracellular calcium increase and akt1/PKB activation that follow the oligomerization of triggering receptors.

## Materials and Methods

### Ethics Statements

Peripheral blood mononuclear cells (PBMC) were obtained from venous blood samples of healthy donors (HD) enrolled in the Vascular Endothelial Growth Factor Study approved by the Ethic Committee of the San Martino Hospital (n.127/06). Patients with chronic lymphocytic leukemia (CLL) were from the Clinical Hematology Department (IRCCS AOU San Martino) and venous blood samples were obtained according to the Ethic Committee of this institute (IRB approval 0026910/07, renewal 03/2009). According to the ethic committee approval, a written informed consent was obtained from all participants.

### Monoclonal Antibodies and Reagents

Anti-CD16 (NK1, IgG1) mAb, anti-CD56 (TA181H12, IgG2a) mAb, anti-CD54 mAb (ICAM-1, M37, IgG1) and the anti-LFA1a (T96H6, IgG1) were obtained in our laboratory as described (29,30). Anti-CD3mAb (Leu4, IgG1), anti-CD4 mAb (Leu3a, IgG1), anti-CD8 mAb (Leu2a, IgG1), anti-CD56 mAb (Leu19, IgG1), anti-2B4 mAb (clone 2-69, IgG2a), anti-DNAM1 mAb (clone DX11, IgG1) and anti-CD107a mAb were from Pharmingen International (San Diego, CA 92121). The anti-NKp30 mAb (clone Z25, IgG1), the anti-NKp44 mAb (clone Z231, IgG1) the anti-NKp46 mAb (clone BAB281, IgG1) as purified azide free were from Immunotech (Marseille, France). The NKG2D mAb (clone 149810, IgG1) was from R&D Systems (Minneapolis, MN 55413) while mevalonate lattone, dimethyl sulfoxide (DMSO) and ethanol were from Sigma (Sigma-Aldrich, Milan, Italy). The affinity purified goat anti-mouse (GAM) anti-isotype specific antiserum was from Southern Biotechnology (Birmingham, AL). Purified GAM anti-Ig(H+L) was purchased from ICN Biomedicals Inc. (Aurora, Ohio 44202). Cells were cultured in RPMI 1640 medium supplemented with 10% of fetal calf serum (FCS), glutamine and penicillin-streptomycin (Biochrom, Berlin, Germany). Atorvastatin, fluvastatin, mevastatin, simvastatin and pravastatin, as well as the calcium-specific chelator BAPTA-AM (1,2-bis-(O-aminopenoxy)-ethane-N, N, N’, N’-tetraacetic acid tetra acetoxymethyl ester) and EGTA were obtained from Calbiochem (EMD Biosciences Inc. San Diego, CA 92121), and used at 0.1-1-10 µM final concentration. Human recombinant interleukin (IL)2, PE or Alexfluor488 or 647 conjugated anti-isotype specific GAM were from Invitrogen (Life Technologies, Monza, Italy). Cholesterol/Cholesteryl Ester Quantitation Kit was from Biovision (Inc. Headquarters Mountain View, California 94043).

### Indirect Immunofluorescence

Single fluorescence staining was performed as described elsewhere [29], [30]. Briefly, aliquots of 10^5^ cells were stained with the corresponding mAb followed by PE- or Alexafluor647-conjugated anti-isotype specific GAM serum or with an unrelated mAb (Pharmingen) followed by the fluorescent second reagent. Samples were analyzed by flowcytometry (CyAn ADP, Beckman-Coulter) equipped with an argon ion laser exciting PE at 488 nm or an He-Ne laser for Alexafluor647 at 613 nm. Data were analyzed using Summit 4.3.01 computer program. Results are expressed as Log red or far red mean fluorescence intensity (MFI) in arbitrary units (a.u.) (x-axis) vs number of cells (y-axis).

### Isolation and Culture of Polyclonal and Clonal NK Cell Populations

NK cells were isolated with the RosetteSep NK isolation kit (Stemcell Biotechnologies, Vancouver, Canada) from heparinized blood of healthy volunteers with slightly modification of the kit protocol. Briefly, PBMC were isolated by Ficoll Hypaque gradient centrifugation and then seeded for two hours at 37°C in plastic Petri dishes at 50×10^6^ cells/plate. Cells were then mixed with autologous red cells at 1∶30 (PBMC:red cells ratio) and Rosettesep NK isolation kit according to manufacturer’s instruction. The resulting cell population was 80–95% CD16^+^ (range of 8 different experiments) but 99% CD3^−^NKp46^+^. These freshly isolated NK cells were immediately used (ex-vivo) or put in culture with 10 ng/ml of IL2 (10^5^ cells/well in 96U-bottomed microplates in 200 µl of complete medium). After 3d or 6d of culture NK cells (short-term activated) were used in functional experiments. NK cell clones were obtained by culturing highly purified CD3^−^ NK cells under limiting dilution conditions with 1 µg/ml of PHA and cultured in 96-well U-bottom microplates (Greiner Labortechnic, Nurtingen, Germany) with RPMI 1640 medium supplemented with 10% of FCS in the presence of 10 ng/ml of interleukin (IL)2 in a final volume of 200 µl/well in the presence of 10^5^/well irradiated autologous PBMC [29,30)]. In particular, decreasing number of CD3^−^ NK cells (100, 50, 25, 12, 6) were seeded in 200 µl/well in 96-well plates. After 10–12d, cells were expanded for additional 30–45d (long-termed activated NK cells). Cloning efficiency was of 5–10% [31]. Cell cultures obtained at 6, 12 and 25 cell/well were then analyzed for the expression of the NK cell specific marker NKp46 with PE-conjugated anti-NKp46 mAb by direct immunofluorescence and only cell cultures homogeneously positive for this receptor were further used in functional assays.

### NK Cell Treatment with Statins

Statins (0.1-1-10 µM ) were added, at the onset of culture, to ex-vivo or short-term (3d or 6d) IL2-activated NK cells. Control cell cultures were represented by NK cells incubated with the solvent of statins (DMSO/H_2_O) and/or of mevalonate lattone (ethanol), 1∶1000 in culture medium, that is the dilution of solvent in 10 µM statin solution. Control samples of NK cells in complete medium were used for comparison with drug- or solvent-treated samples. In some experiments untreated or statin-treated NK cells were incubated for 45 min at 37°C with the calcium-specific chelator BAPTA-AM at 32.7 µM final concentration.

### Cytolytic assay, CD107a Surface Expression and BLT Esterase Assay

Cytolytic activity of ex-vivo or short-term IL2 activated NK cells or NK cell clones was analyzed in a 4 h ^51^Cr-release assay (in some experiments a 24 h assay was performed) against a panel of tumor target cells including the melanomas FO1 and Mel10538, the lung adenocarcinoma A549, the erythroleukemia K562, the myeloid leukemias cell line U937 and HL60, the CD3^+^ T cell lymphoma Jurkat cell line, the CD20^+^ Burkitt B lymphoma cell line Raji and the C1R lymphoma cell line (ATCC, USA). The breast adenocarcinoma HER2^+^ cell lines BT474 and SKBR3 (ATCC, USA), or the CD20^+^ C1R cell line were used to evaluate the effect of statin treatment on ADCC mediated by the anti-HER2 (trastuzumab) or anti-CD20 (rituximab) antibody respectively. These antibodies were used at 20-2 and 0.2 µg/ml and within this range of concentration no statistically significant differences were found. Target cells were labelled with ^51^Cr and used at an effector target (E:T) ratio of 20∶1. One hundred microliter of supernatant were counted in a γ-counter and % of ^51^Cr specific release was calculated as described [30], [31]. In the re-directed killing assay the FcγR^+^ murine mastocytoma cell line P815 was used as target in the presence of optimal amounts (1–5 µg/ml) of the indicated mAb (30,31) or in medium alone at the indicated E:T ratio determined in preliminary experiments. It is of note that the same degree of CD16-mediated activation was achieved with 1 µg to 0.001 ng/ml of anti-CD16 mAb. Expression of CD107a at the NK cell surface was analyzed as described [32]. Secretion of granzymes was analyzed by BLT esterase assay as previously reported [33], [34].

### Cholesterol Quantification

Highly purified ex-vivo NK cells were incubated with fluvastatin or pravastatin (0.1-1-10 µM) for 72 h with IL2. NK cells incubated with the solvent of either fluvastatin (DMSO) or pravastatin (H_2_O) at the same molar concentration as in statins incubated samples were the controls of statins treated cultures. At the end of the treatment cholesterol/cholesteryl ester was quantified using colorimetric methods according to the kit instruction (Biovision). Results are shown as µg/10^6^cells.

### Calcium Mobilization Assay

To evaluate the kinetics of intracellular free calcium increase ([Ca^2+^]_i_), NK cells were loaded for 1 h at 37°C with the calcium probe Fura-2AM (1 µM, Invitrogen), washed and stained with antibody to a given receptor at 4°C and then [Ca^2+^]_i_ analyzed after the addition of GAM to achieve optimal cross-linking. Samples were placed in a 1 cm quartz cuvette and Fura2 was excited at 340 nm and 380 nm; emitted light was filtered at 510 nm and fluorescence was monitored with the spectrofluorimeter LS50B (Perkin Elmer, Ponoma, CA). The ratio of 340/380 nm excitation Fura-2 emitted fluorescence at 510 nm was analyzed and nM [Ca^2+^]_i_ calculated by the computer program FLWinLab 3.0 (Perkin Elmer). To determine the percentage of responding cells to a given stimulus NK cells were labelled with 1 µM Fluo-4 (Invitrogen) green fluorescent probe for 30 min at 37°C in calcium assay buffer. After extensive washes, cells were incubated at 4°C for 30 min with mAbs (3 µg/ml) to surface receptors indicated in each figure, washed and run on CyAn ADP flow cytometer at time 0 (basal) or after cross-linking of the corresponding activating receptor with 10 µg/ml GAM at 1 min, 5 min and 10 min. As the fluorescence of a given cell sample was proportional to Ca^++^ combined with Fluo-4, the ratio between F_1_ (fluorescence after cross-linking) and F_0_ (fluorescence at time 0) is a measure of the increment of intracellular free Ca^++^ concentration [Ca^++^]_i_. Results presented are referred to Fluo-4 fluorescence at 10 min as in kinetics experiments this was the time point at which stimulation had reached the maximal level. The percentage of responding cell was determined by gating NK cells on the basis of FSC and Fluo-4 staining, excluding debris and dead cells.

### TNFα Production and FasL Release

10^6^ NK cells were incubated at 4°C for 30 min with mAbs (3 µg/ml) to activating surface receptors, washed and incubated for additional 24 h at 37°C in complete medium in 24w plates coated with GAM to obtain cross-linking of a given receptors. In some experiments NK cells were incubated with tumor target cells at 5∶1 E:T ratio for 24 h. Supernatant (SN) were harvested and analyzed for the presence of FasL and TNFα by specific ELISA (Peprotech EC Ltd London, UK) for TNFα or home-made for FasL [34]. Results are shown as pg/ml/10^6^ cells. In other experiments, NK cells were treated with BAPTA-AM at 32.7 µM and incubated for 24 h at 37°C with Jurkat target cells at 2∶1 E:T ratio. The cytotoxic effect of TNFα was assessed using the TNFα sensitive cell line WEHI164 either in the absence or presence of anti-TNFα polyclonal antibody (Peprotech).

### Confocal Microscopy

For the analysis of actin distribution after NK cell triggering via LFA1, NK cells were cultured in the presence of fluvastatin, washed and put on glass cover slips at 37°C for 20 min coated with the ICAM1 molecule [6]. Samples were fixed with 3.7% paraformaldeide, permeabilized with 1∶100 NP-40 and stained with Alexa-488 phalloidin which binds to actin filaments. Ganglioside M1 rafts were detected on NK cells treated with fluvastatin upon engagement of different surface receptors at 37°C with the specific antibody followed by GAM and staining with cholera toxin subunit A conjugated with Alexafluor488. In some experiments, NK cells cultured as described were stained with anti-perforin mAb (IgG2b) and anti-FasL mAb (Alf1.2, IgG1, Sigma) or anti-calnexin mAb (AF18, IgG1, Abcam, Cambridge, UK) as a marker of endoplasmic reticulum or anti-CD107a mAb (H4A3, IgG1, Pharmingen) followed by anti-isotype specific Alexafluor488 or 647 GAM. Analysis was performed with the confocal microscope FV500, Olympus IX81, equipped with oil objectives PlanAPO 40x and 60x NA 1.40 and images were taken with the computer program FluoView 2.1. Images were analyzed with the analysis FIVE computer program (Soft Imaging System GmbH,Munster, Germany) to enumerate the % of NK cells with rafts or granule containing FasL and/or perforin.

### Analysis of Activation of RhoA

The level of activation of RhoA GTP-binding protein was analyzed on NK cells after LFA1 cross-linking. Briefly, 4×10^6^ NK cells for each time point were incubated with anti-LFA1 mAb for 30 min at 4°C and then cross-linked with GAM at 37°C in complete medium. Activation of RhoA was analysed using the RhoA activation kit (RhoA G-lisa kit, Cytoskeleton, Tebu-Bio, Milan, Italy) at 5 min, 12 min and 30 min after cross-linking and each sample was processed as indicated by manufacturer’s instruction. Each sample was performed in triplicates and the activation of RhoA in LFA1 cross-linked samples was evaluated in comparison to the level of activation in the absence of cross-linking. In parallel experiments, the actin distribution in a given cell was analyzed by confocal microscopy, after cross-linking of LFA1, with Alexa488 phalloidin (Invitrogen).

### Analysis of Activation of akt1/PKB

The activation of the serine/threonine kinase akt1/PKB in cell lysates of NK cells was assessed with a commercial ELISA assay kit (Upstate Biotechnology, Lake Placid, NY). Akt1/PKB activity was tested upon ligation of surface receptors (CD16 or NKG2D) and the protein content in each sample was normalized. The amount of total akt1 and phosphorylated akt1was evaluated with specific antibodies at least in triplicate and results are expressed as % of akt1/PKB phosphorylation that is the ratio of phophorylated akt1 and total akt1. The activation of akt1/PKB is dependent to the phosphoinositide kinase 3 (PI3K) thus some experiments were performed in the presence of the PI3K inhibitor LY294002 to confirm the involvement of this kinase [36].

### Statistical Analysis

Results obtained from at least 6 different experiments for each experimental setting were analyzed by applying paired two tail student t test at 95% confidence.

## Results

### Fluvastatin Impairs the Activation of the Small GTP Binding Protein RhoA, Actin Distribution and Ganglioside M1 Rafts Formation in NK Cells

**Figure 1:**
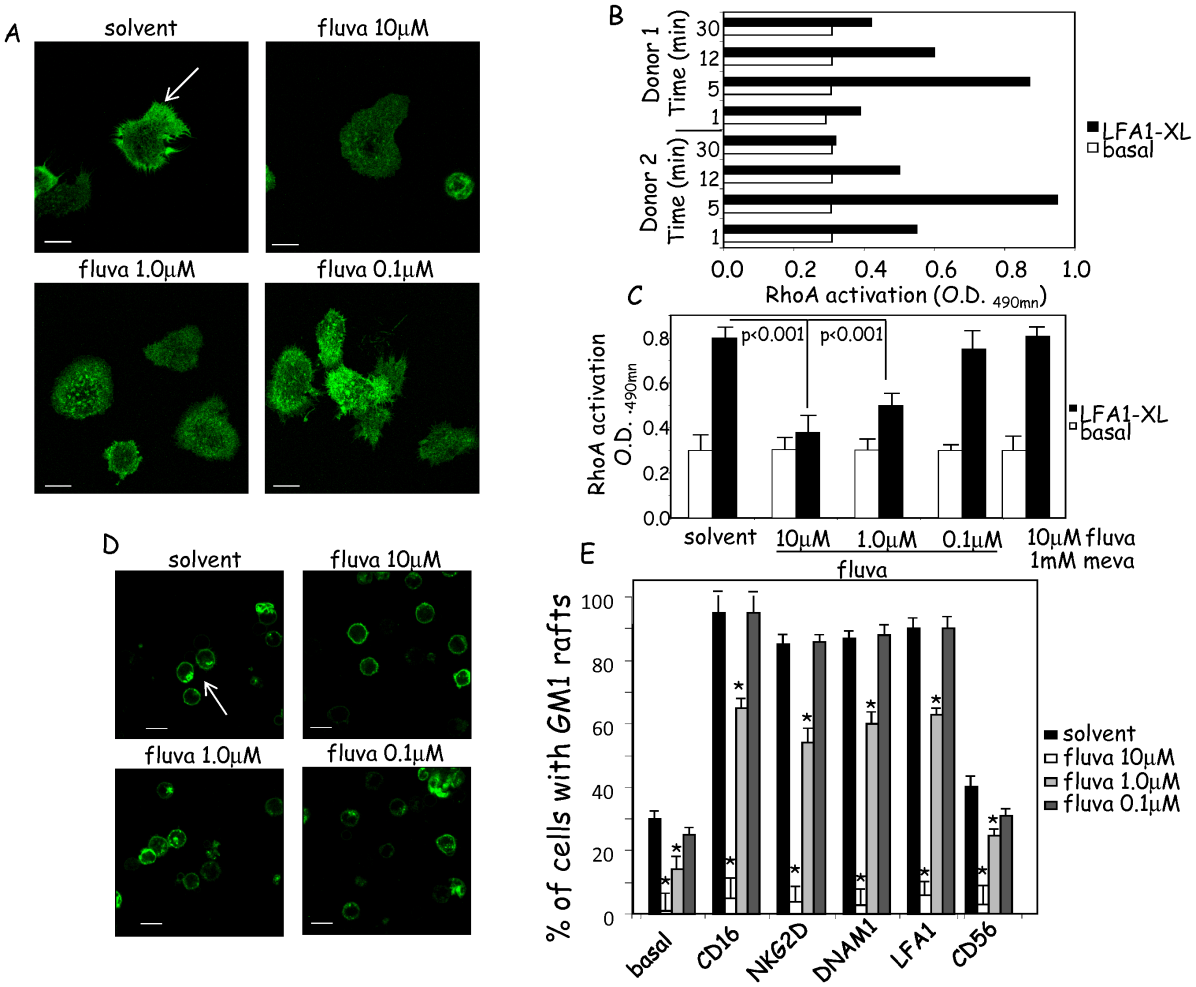
Actin rearrangement and Rho A activation upon LFA1 engagement in NK cells. (A). Rearrangement of actin microfilaments was analyzed in NK cells cultured in the indicated conditions (solvent of fluvastatin, DMSO: solvent; or fluvastatin at different doses 10-1.0-0.1 µM) for 6d in the presence of IL2 (10 ng/ml). LFA1 crosslinking was induced on ICAM1 protein coated slides for 20 min at 37°C. Then cells were fixed and permeabilized and incubated with alexafluor 488-phallotoxin to stain actin. Bar in each subpanel: 5 µm. Samples were analyzed by confocal microscopy (Olympus FluoView 500) and images were taken with PlanApo objective 60x/1.20NA and analyzed with FluoView computer program. Actin rearrangement is characterized by well-marked lines more evident at the periphery of a cell (A, upper left, white arrow). No actin rearrangement in NK cells treated with fluvastatin at 10 µM (A, upper right). Results are representative of three independent experiments with NK cells from different donors. (B). Kinetic of RhoA activation by specific C-Lisa kit. Black bars: LFA1 engagement using anti-LFA1mAb followed by GAM (LFA1-XL), white bars level of RhoA activated in basal conditions at the indicated time points from two healthy donors. (C). Activation of RhoA on NK cells cultured in the solvent of fluvastatin (solvent) or with the indicated doses of fluvastatin (10-1.0-0.1 µM) or with 10 µM fluvastatin and 1 mM mevalonate. Results in panels B and C are expressed as OD at 490 nm from two donors (B) or mean±SD of data from six different donors (C). (D,E): effect of fluvastatin on membrane rafts containing GM1. NK cells cultured 6d in IL2 with solvent (DMSO) or fluvastatin (10-1.0-0.1 µM) were incubated with anti-NKG2D mAb followed by GAM for 20 min and stained with alexafluor 488 subunit A of cholera toxin which reacts with GM1. Membrane rafts with GM1 were displayed as patches (D, white arrow). (E). Rafts with GM1 in NK cells incubated with the indicated mAbs followed by GAM. Basal: rafts in NK cells without any cross-linking. Results are shown as % of cells with GM1 rafts for each culture conditions. At least 200 cells were counted for each experiments and results are the mean±SD of six independent experiments.

It has been reported that lipophilic statins affect granule polarization at the site of contact between NK and tumor cells suggesting a possible inhibition of cytoskeleton reorganization and LFA1-mediated triggering [26]. To clarify the molecular mechanism involved, we analyzed the effect of fluvastatin on actin distribution, on activation of the small GTP-binding protein RhoA needed for actin assembling [37], [38], and on ganglioside M1 raft formation in NK cells. In ex-vivo isolated or IL2-activated (6d) NK cells actin was not assembled beneath the plasma membrane but dispersed in the cytoplasm and not easily detectable (not shown). To stimulate actin assembling, NK cells cultured for 6d with IL2, with or without fluvastatin at different doses (fig. 1A), were harvested and the engagement of LFA1 was achieved using glass slides coated with purified ICAM1. A 15–30 min incubation at 37°C, led to actin redistribution, evidenced by detectable lines beneath the membrane marking the limit of each NK cell (fig. 1A, upper left). The assembling and redistribution was markedly reduced in NK cells treated with 10 µM fluvastatin (fig. 1A, upper right). This inhibiting effect was less evident at 1 µM and undetectable at 0.1 µM concentration. In parallel experiments, we found that the kinetics of RhoA activation upon cross-linking of LFA1 peaked at 5 min (fig. 1B), but strikingly decreased in NK cells cultured with fluvastatin (fig. 1C: 15%, 30% and 70% RhoA activation in 10-1-0.1 µM fluvastatin respectively vs untreated cells). The addition of mevalonate together with fluvastatin reverted the effect of the statin (fig. 1C). Then, we analyzed whether fluvastatin can affect the formation of membrane rafts containing ganglioside M1 [39], [40]. No rafts were found in ex-vivo isolated NK cells and upon culture with IL2, 20–30% of NK cells displayed rafts (fig. 1E). Engagement of the NK cell surface activating receptors NKG2D (fig. 1D,E) or CD16 or DNAM1 or LFA1 (fig. 1E) triggered the formation of GM1 rafts in almost all NK cells (90–100%). These rafts were not present in NK cells treated with 10 µM fluvastatin; at 1.0 µM fluvastatin, we found about 70% of NK cells with rafts, while no effect was detected at 0.1 µM concentration. In NK cells cultured with 1 mM mevalonate lattone the inhibiting effects of fluvastatin on RhoA activation and on GM1 rafts were abolished (not shown).

Experiments demonstrating that fluvastatin inhibits NK cell-mediated lysis of tumor cells elicited both upon LFA1-ICAM1 interaction and upon triggering through specific activating receptors are described in File S1 and depicted in Figure S1, S2, S3.

### Fluvastatin Inhibits the Triggering of Receptor-mediated Intracellular Free Calcium Increase and akt1/PKB Activation in NK Cells

**Figure 2:**
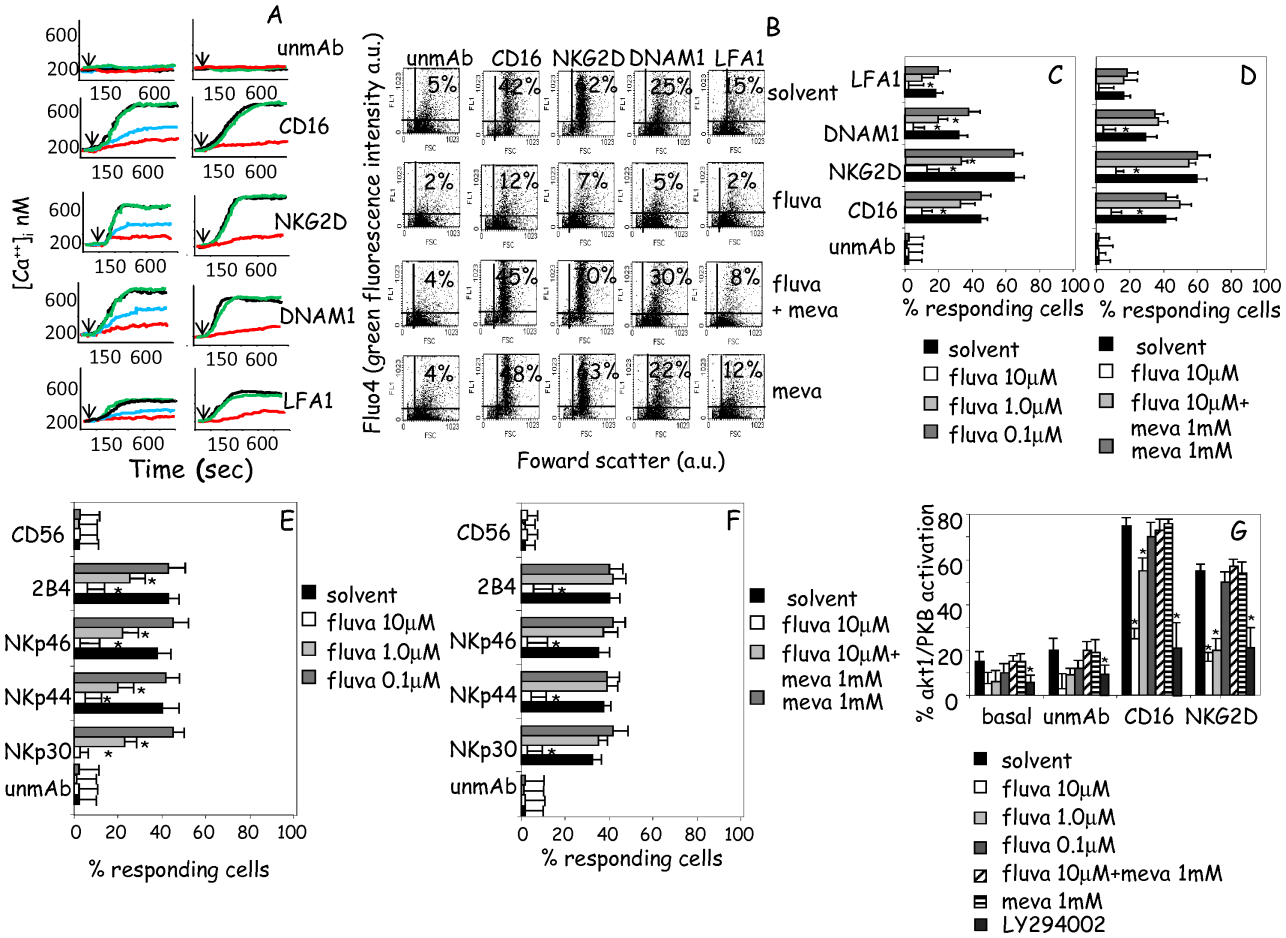
Fluvastatin effects on intracellular calcium increase and akt1/PKB activation. (A–F) Fluvastatin inhibits intracellular free calcium increase [Ca^2+^]_i_ induced upon engagement of NK cell activating receptors. (A). Effect of fluvastatin on calcium increase triggered by CD16 or NKG2D or DNAM1 or LFA1 with the specific mAbs followed by GAM; unmAb: unrelated mAb matched for isotype plus GAM. NK cells were cultured without drug (black), with fluvastatin (10 µM, red; 1 µM, blue; 0.1 µM, green; left panels) or in right subpanels with fluvastatin alone (red, 10 µM) or in combination with 1 mM of mevalonate (green) for 6d+IL2. Then cells were harvested, washed and labelled with the fluorescent calcium indicator Fura-2 at 37°C and [Ca^2+^]_I_ analyzed on a spectrofluorimeter along time (800 sec). The arrows indicate the addition of GAM to achieve the optimal cross-linking for the indicated receptors. (B) Effect of fluvastatin on calcium increase triggered as in A. NK cells were cultured without drug (solvent), with fluvastatin alone (10 µM, fluva) or in combination with 1 mM of mevalonate (fluva+meva) or with mevalonate alone (meva) for 6d+IL2. Then cells were harvested, washed and labelled with the fluorescent calcium indicator Fluo-4 for 45 min at 37°C; intracellular free calcium increase was assessed by flowcytometry upon engagement of the indicated surface receptors after 600 sec of incubation. Results are shown as forward scatter (FSC, x-axis) vs Log green fluorescence intensity (Fluo-4, Y-axis). Each subpanel is divided in four quadrants: lower left: very small cells/debries; lower right: cells with FSC of living NK cells; upper left: small cells/debries labelled with Fluo-4; upper right: NK cells strongly labelled with Fluo-4. In each subpanel in the upper right quadrant is shown the % of NK cells with high Fluo-4 fluorescence. Evaluation of Fluo-4 staining was performed after 10 min upon engagement of each molecule. (C–F). Percentage of NK cells (% responding cells), cultured with different doses of fluvastatin (C,E) or different combination of drugs (D,F), with an increase of intracellular calcium concentration upon engagement of the indicated receptors. Results are expressed as mean of responding cells ±SD of 6 independent experiments. *p<0.001. (G). Evaluation of activation of akt1/PKB in NK cells cultured as in A–F. Results are expressed as % of akt1/PKB activation as ratio between pakt1 and total akt1×100 in each condition at 5 min after stimulation with mAb to the indicated receptors and GAM. Basal: basal level of akt1/PKB activation in cultured NK cells. LY294002: akt1/PKB activation in the presence of the upstream PI3K inhibitor LY294002. *p<0.001 vs solvent.

As intracellular free calcium [Ca^2+^]_i_ mobilization and phosphoinositide kinase 3 (PI3K) activation are crucial for the release of cytotoxic granules [8], [33], [36], we analyzed whether the inhibition of cytolytic activity mediated by fluvastatin was related to impaired [Ca^2+^]_i_ increase and/or inhibition of PI3K-dependent activation of akt1/PKB in NK cells. To this aim, [Ca^2+^]_i_ increase in NK cells cultured with IL2 for 6d with scalar doses of fluvastatin (10-1.0-0.1 µM, fig. 2A left subpanels) was analyzed on a spectrofluorimeter upon the engagement of CD16, or NKG2D or DNAM1 or LFA1 receptors after labelling of cells with Fura-2 calcium probe. We found that fluvastatin exerted a strong and dose dependent inhibiting effect (fig. 2A left panels) that was reverted by the addition during the culture of mevalonate (fig. 2A, right panels). Furthermore, to determine whether the observed inhibiting effect was dependent on the amount of responding cells, primary NK cells cultured with IL2 for 6d without or with fluvastatin, were washed and labelled with Fluo-4 calcium probe. The increase in the intensity of fluorescence of cell samples due to [Ca^2+^]_i_ increase was analyzed by flowcytometry after 600 sec, either without or upon cross-linking of the same triggering receptors. The engagement of the activating receptors CD16, NKG2D, DNAM1 (fig. 2B–D) or NKp30, NKp44, NKp46 or 2B4 (fig. 2D,E) induced a strong [Ca^2+^]_i_ increase while cross-linking of LFA1 was less efficient (fig. 2A–C). NK cells cultured with fluvastatin showed a lower [Ca^2+^]_i_ rise (fig. 2B) and this effect was dose dependent (fig. 2C,D). We found a lower number of responding NK cells in cell cultures with 10 µM than with 1 µM fluvastatin (fig. 2C,E); at 0.1 µM fluvastatin no effect was observed (fig. 2C,E). The addition of mevalonate at the onset of cell culture completely restored the response to the triggering receptors in fluvastatin-treated NK cells (fig. 2D,F). Further, we found that the engagement of CD16 or NKG2D induced a marked activation of akt1/PKB; this activation was inhibited in NK cells treated with LY294002 indicating the involvement of the upstream PI3K (fig. 2G). Activating receptor-mediated triggering of akt1/PKB was impaired in NK cells incubated with fluvastatin; it is of note that CD16-mediated phosphorylation was less inhibited than that elicited upon NKG2D cross-linking (fig. 2G). Again, NK cells cultured with fluvastatin and mevalonate showed a response to these triggering receptors similar to untreated NK cells (fig. 2G).

### Fluvastatin Inhibits Perforin and Granzyme Release but not FasL Secretion in NK Cells

**Figure 3:**
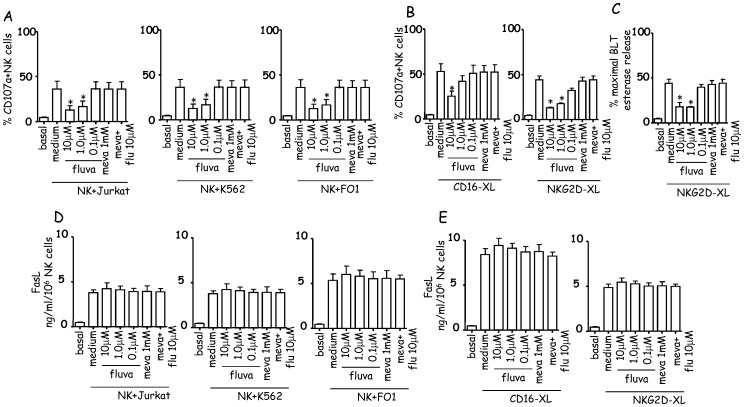
Fluvastatin selectively inhibits the release of perforins and granzymes but not of FasL. NK cells were culture for 6d+IL2 with the indicated doses of drugs or in their solvent. Then NK cells were co-cultured with Jurkat or K562 or FO1 (A), (NK/tumor target ratio 2∶1) or incubated with mAbs to CD16 or NKG2D (B) followed by GAM. After 2 h, NK cells were labelled with anti-CD107a mAb and analyzed by flowcytometry. Basal: % of CD107a^+^ NK cells before interaction with tumor cells (A) or engagement of the indicated receptors (B). Results are expressed as mean±SD of % CD107a^+^ NK cells from six different donors. (C). BLT esterase activity of SN from NK cells after the engagement of NKG2D. Results are expressed as % of maximal BLT esterase content in NK cells. Statistical significance *p<0.001 vs medium. (D). Evaluation of soluble (s)FasL in SN from cultures of NK cells and Jurkat or K562 or FO1 tumor cell lines (NK/tumor cell ratio 2∶1 at 8 h) or upon the engagement of CD16 or NKG2D (E) receptors with specific mAb and GAM (at 8 h). Data are shown as ng/ml/10^6^ NK cells and are the mean±SD of 6 experiments.

Since NK cells can kill tumor targets either through perforin-mediated cytotoxicity or following FasL secretion, we analyzed whether fluvastatin can impair both perforins-granzymes and FasL release. CD107a surface expression on NK cells was used to indirectly evaluate perforin release [32] either after interaction with target cells or upon cross-linking of activating NK receptor with specific mAb and GAM (fig. 3A,B). Fluvastatin impaired CD107a expression at the NK cell surface upon incubation with Jurkat or K562 or FO1 tumor cell lines (fig. 3A). Also, CD107a surface expression induced by engagement of either CD16 or NKG2D was consistently reduced in fluvastatin cultured NK cells (fig. 3B). However, upon CD16 engagement, inhibition was statistically significant when NK cells were cultured with 10 µM but not 1 µM of fluvastatin, while NKG2D-mediated triggering was significantly inhibited at both doses. The low amount of BLT esterase activity elicited upon NKG2D engagement and found in supernatants from fluvastatin-treated NK cells, compared with the high BLT esterase activity found in medium (fig. 3C) or solvent-treated (not shown) NK cells, confirmed that fluvastatin impairs NK cell degranulation. The addition of mevalonate together with fluvastatin completely reverted the effect of fluvastatin (fig. 3A–C). In parallel samples, the release of FasL in culture supernatant was evaluated by ELISA. We found that the interaction of NK cells with Jurkat or K562 or FO1 target cells triggered the release of similar amounts of FasL from NK cells cultured either in medium or with different doses of fluvastatin (fig. 3D). Also, triggering of NK cells by cross-linking of either CD16 or NKG2D could induce the secretion of FasL and, again, fluvastatin-treated NK cells could release FasL in similar amount as untreated (fig. 3E) or solvent treated (not shown) NK cells.

These findings suggest that the secretion of granules containing perforin/granzymes is inhibited in fluvastatin-treated NK cells while the secretion of FasL is not affected. To explain this difference, we analyzed the subcellular localization of perforins and FasL in NK cells. We found that in IL2-cultured NK cells perforins are localized in discrete granules while FasL shows a distribution similar to that of the endoplasmic reticulum marker calnexin (fig. S4 panel A). Furthermore, we found that upon cross-linking of the activating receptor NKG2D we could observe in NK cells granules containing either perforin or FasL or granules with both perforin and FasL (fig. S4 panel B,C). Indeed, the large majority of granules contained either one or another cytotoxic factor (60-85%) while a minority (40–15%) contained perforin together with FasL (fig. S4 panel D).

### Fluvastatin does not Inhibit FasL/Fas–mediated Killing of Tumor Cells

**Figure 4:**
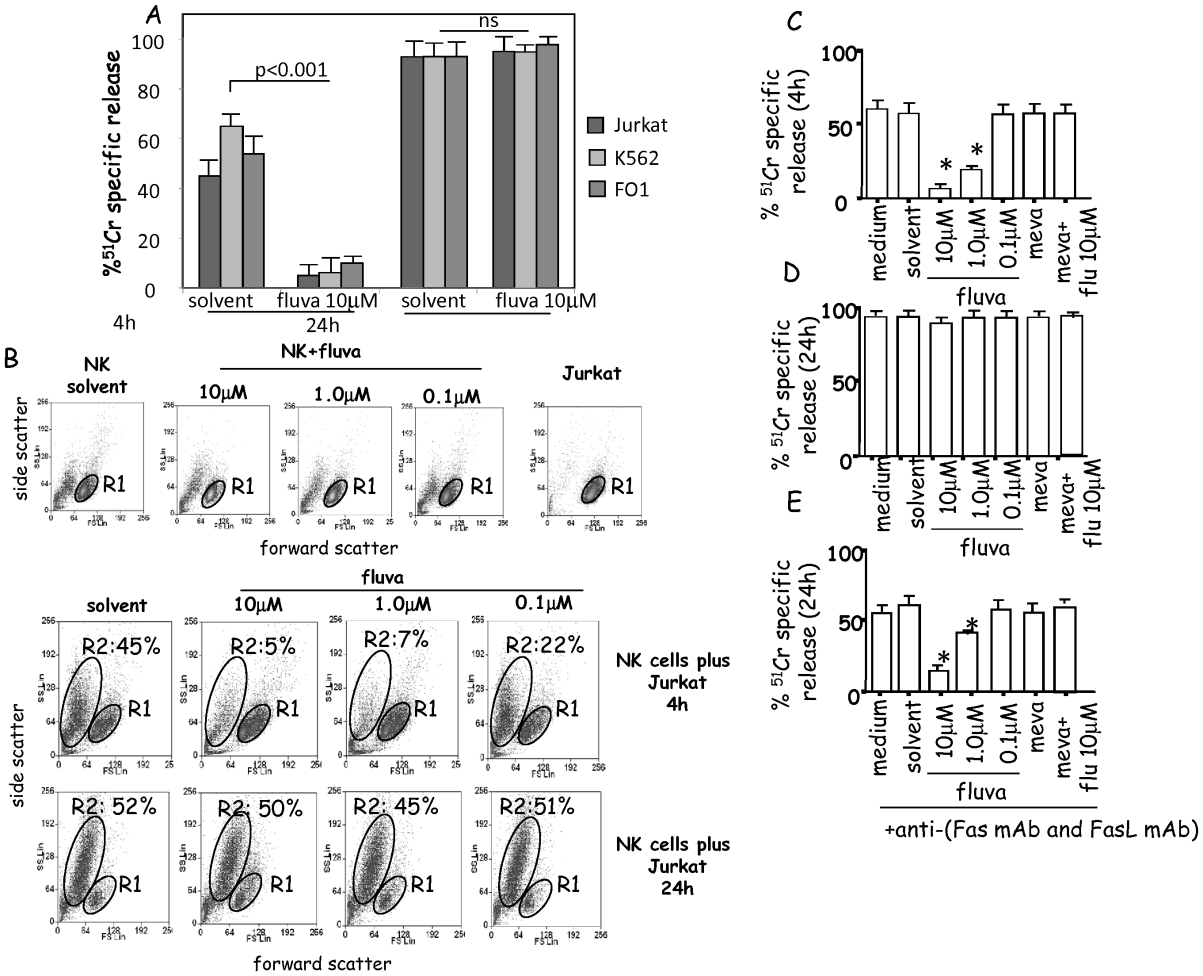
Fluvastatin does not inhibit NK cell mediated cytotoxicity of target cells mediated by FasL/Fas receptor ligation. (A). Cytotoxic activity of NK cells cultured with solvent or fluvastatin 10 µM for 6d+IL2 was analyzed after 4 h or 24 h incubation with the indicated target cells (Jurkat, K562 or FO1). Results are expressed as percentage of ^51^Cr specific release and are the mean±SD of six experiments. (B, first row). Side scatter (SSC) and forward scatter (FSC) of ex-vivo isolated NK cells cultured for 6d+IL2 in solvent (DMSO) or in presence of fluvastatin (10-1.0-0.1 µM). FSC and SSC of Jurkat cell line is also shown. R1 gate: living cells. (B, second and third row) NK cells, in the indicated culture conditions, were co-incubated with Jurkat cells for 4 h (second row) or 24 h (third row) at 2∶1 E:T ratio. R1 gate: living cells; R2 gate: dying cells. Results are representative of three independent experiments and number in panels indicate the % of R2 gated cells. (C–E). NK cells were cultured for 6d+IL2 in medium alone (medium) or solvent of fluvastatin (solvent) or with fluvastatin (10-1-0.1 µM) or with mevalonate 1 mM either alone or with fluvastatin 10 µM as indicated. NK cell-mediated cytotoxicity of Jurkat cells at 2∶1 E:T ratio analyzed after 4 h (C) or 24 h (D) without mAbs added; (E): anti-FasL and anti-Fas mAbs added at the onset of the 24 h cytotoxicity assay.

Cytolysis that occurs upon polarized release of granules containing perforin and granzymes leads to necrosis of target cells in a short time, usually detected after 4 h of incubation, while FasL induces apoptosis of targets after more than 8 h, being detectable at 24 h [1]–[5], [29], [30]. Thus, we analyzed whether fluvastatin can differently affect these two mechanisms of killing. We found that NK cell-mediated cytotoxicity of Jurkat, K562 or FO1 tumor targets strongly increased when analyzed after 24 h compared to the killing detected at 4 h (fig. 4A). At 4 h, NK cells incubated with fluvastatin exerted a lower cytotoxic activity vs Jurkat or K562 or FO1 compared to that of NK cells cultured in medium (not shown) or solvent (fig. 4A). Conversely, at 24 h, we found that the killing of the same targets was not inhibited by fluvastatin (fig. 4A). Focusing on Jurkat cells, which are sensitive to Fas/FasL mediated apoptosis [29], [30], we analyzed more in detail the mechanism of cytotoxicity at 24 h, that was not inhibited by fluvastatin. First, we found that the shape and size of cells analyzed as forward scatter (FSC) and side scatter (SSC) in NK/Jurkat cell co-cultures were not altered at 4 h when NK cells were incubated with fluvastatin (a few cells present in the region R2 of fig. 4B plots at 4 h). On the contrary, FSC and SSC of co-cultures changed at 24 h (large amount of cells in R2 region, fig. 4B). Accordingly, killing of Jurkat cells was inhibited in fluvastatin treated cells at 4 h (fig. 4C) but not at 24 h (fig. 4D). Furthermore, the addition (after 4 h from the onset of the cytotoxicity assay) of anti-Fas and anti-FasL mAb, to block the interaction between FasL secreted by NK cells and Fas molecule on target cells, markedly inhibited the killing detected at 24 h suggesting that it is Fas/FasL-dependent (fig. 4E). It is of note that the degree of killing of Jurkat cells chelating extracellular calcium with EGTA, and evaluated after 24 h, was superimposable using as effector cells either untreated or fluvastatin treated NK cells (not shown). This would suggest that long-term target cell lysis is not dependent on extracellular calcium.

### NK Cells Incubated with Fluvastatin can Still Secrete TNFα

**Figure 5:**
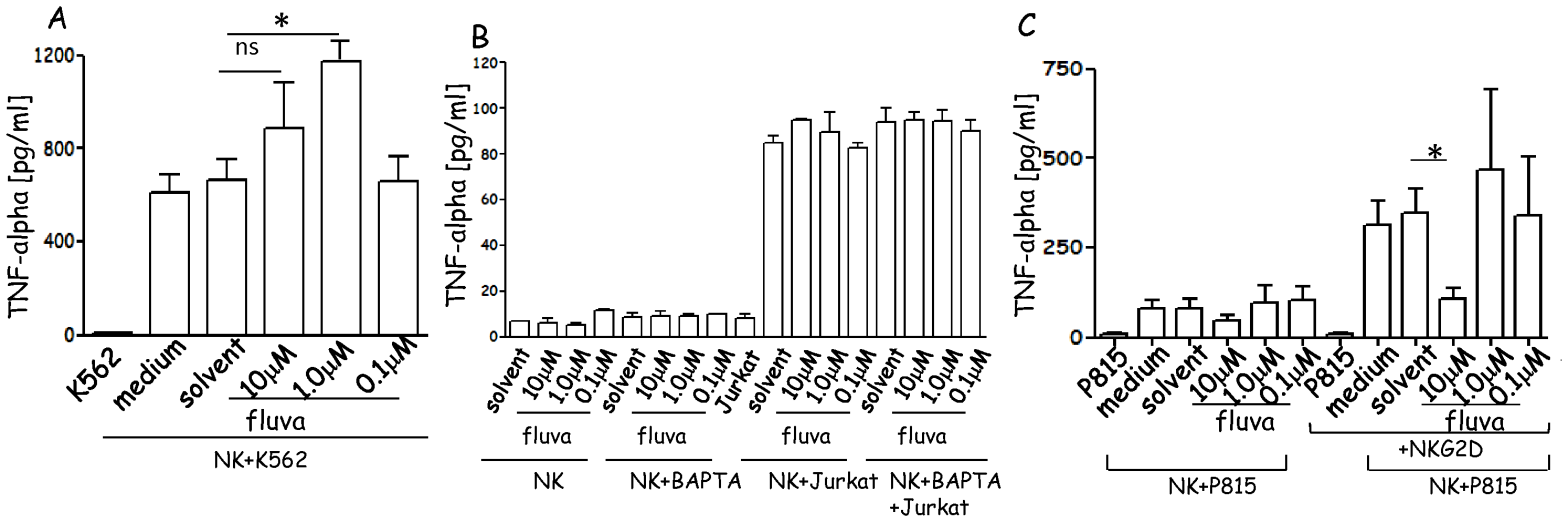
Fluvastatin does not affect TNFα release in NK cells. (A). TNFα, measured by ELISA, present in supernatant of NK cells cultured for 6d+IL2 in medium alone (medium) or solvent of fluvastatin (solvent) or with fluvastatin (10-1.0-0.1 µM) and incubated with K562 HLAI^−^ target cells, at the NK:target cell ratio of 2∶1. (B). TNFα in SN of NK cells, cultured in A, incubated with HLAI+ Jurkat target cells (2∶1 ratio). (C). TNFα in SN of NK cells, cultured as in A, incubated with P815 target cells in the presence of anti-NKG2D mAb to evaluate receptor-mediated TNFα release. In the different panels: K562 (A), Jurkat (B), P815 (C): basal level of cytokine production of tumor target cells. Left column in panel C indicate the basal level of cytokine production of NK cells. Results are expressed as pg/ml and are representative of three independent experiments. In some experiments, the effect of chelation of intracellular calcium with BAPTA-AM on TNFα release was analyzed (B).

To determine whether secretion of TNFα, another mechanism of NK cell-mediated tumor cell killing (1–5), can be regulated by fluvastatin, we analyzed the content of TNFα in the supernatants of NK cells cultured with two targets expressing or not HLAI: a) HLAI^−^ K562 cells (fig. 5A); b) HLAI+ Jurkat target cell (fig. 5B). Indeed, activation of NK cells can be negatively regulated by interaction with HLAI and some tumor can downregulate HLAI to escape immune system, thus it is relevant to determine whether fluvastatin can have different effects. Ex-vivo isolated NK cells were cultured for 6d in IL2-containing medium in the absence or presence of solvent or fluvastatin before co-incubation with K562 cells. Interaction of NK and K562 cells triggered the release of TNFα; more importantly, TNFα was found in culture supernatants also when NK cells were pretreated with fluvastatin (fig. 5A). The amount of TNFα found in NK-K562 co-cultures from NK cells incubated with 1.0 µM fluvastatin was about two-fold than that detected using untreated NK cells (p<0.01). When Jurkat cells were used as targets, TNFα release was lower than that elicited upon incubation with K562 cells (compare fig. 5A and fig. 5B) and fluvastatin did not affect TNFα production. These findings suggest that HLAI can indeed deliver a negative signal in NK cells leading to low secretion of TNFα but again fluvastatin is not active. Furthermore, to define the role of intracellular calcium in the production of TNFα NK cells were incubated with BAPTA-AM calcium chelator, washed and then co-cultured with Jurkat cells. Of note, TNFα secretion was not altered after chelation of intracellular calcium either in the presence or absence of fluvastatin (fig. 5B) indicating that calcium is not mainly involved in the secretion of this cytokine.

To analyze whether the secretion of TNFα induced by a specific triggering receptor was affected by fluvastatin, we incubated NK cells with P815 target cells in the presence of mAb to NKG2D. We choose this triggering molecule as redirected killing of P815 cell line induced via NKG2D was strongly inhibited by 10 µM and 1.0 µM fluvastatin (fig. S2 panel B). Secretion of TNFα elicited through NKG2D was not detectable at 1.0-0.1 µM concentration (fig. 5C) but higher doses as 10 µM of fluvastatin display a significant inhibitory effect (p<0.01 vs basal secretion of TNFα). This indicates that also the sensitivity to fluvastatin.

### Effect of Fluvastatin on ADCC and TNFα Release Mediated through the Engagement of FcγRIIIA with Humanized Antibodies

**Figure 6:**
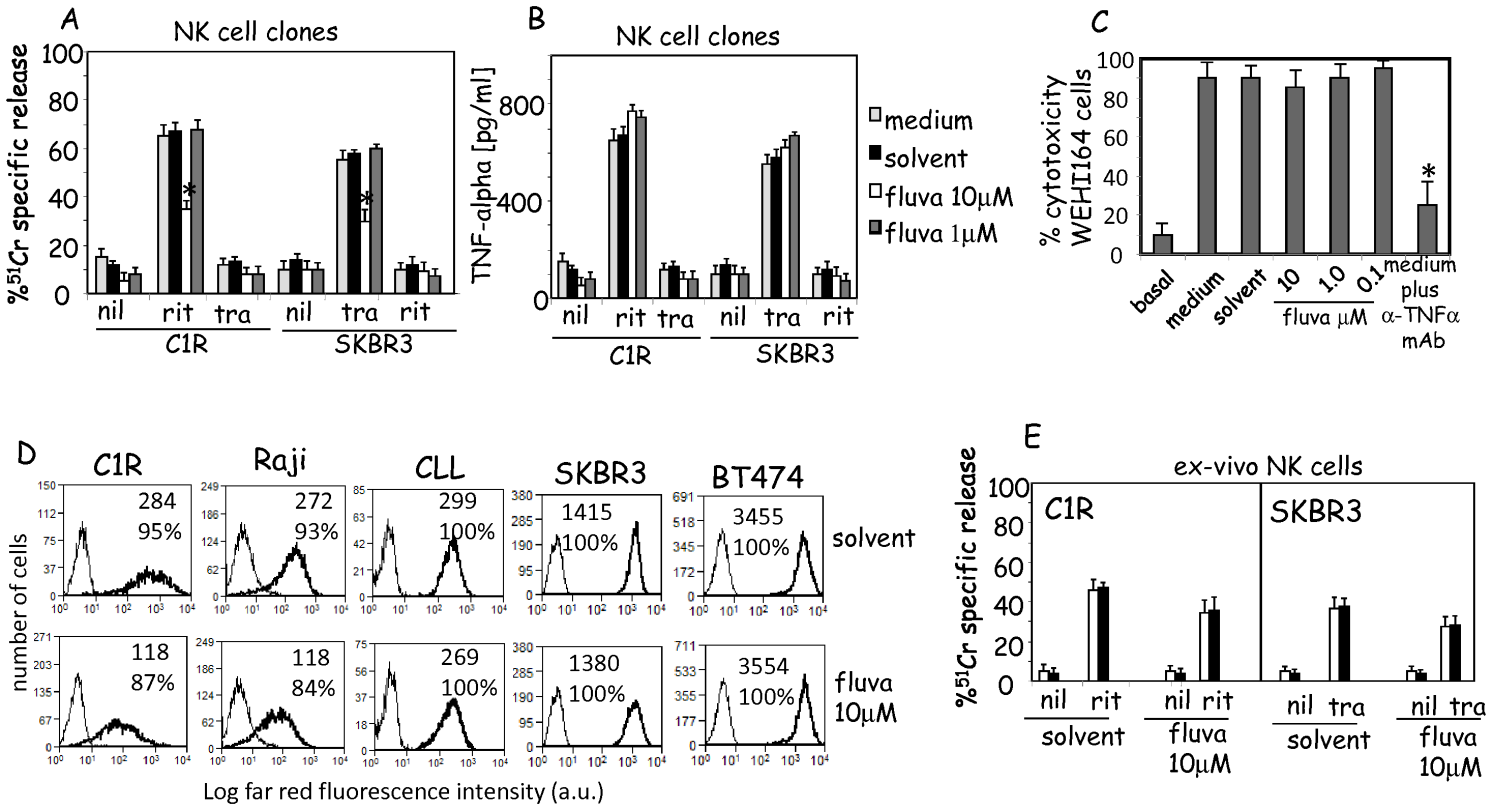
Effect of fluvastatin on ADCC and TNFα release triggered with rituximab or trastuzumab. (A). Effect of fluvastatin on ADCC triggered with humanized antibodies. Cytolytic activity of NK cell clones was analyzed upon treatment with fluvastatin in ADCC assay with rituximab (rit) or trastuzumab (tra) using the C1R CD20^+^ or SKBR3 HER2^+^ tumor cell lines at 2∶1 NK/tumor cell ratio respectively. Nil: basal cytolysis in absence of humanized antibody. Results are expressed as %^51^Cr specific release and are the mean±SD of 30 NK cell clones tested from six donors. (B). TNFα released in the NK-tumor cell co-culture after 24 h incubation was assessed by ELISA. Nil: basal level of TNFα produced by unstimulated NK cell clones. Results are expressed as pg/ml and are the mean±SD of TNFα present in SN from 15 NK cell clones. (C). Cytotoxic effect of TNFα released by NK cells in co-cultures with CIR and rituximab or with SKBR3 and trastuzumab antibody. Cytotoxicity was assessed on WEHI164 cell line sensitive to TNFα-mediated killing. In some experiments to block the cytotoxic effect of TNFα a saturating amount of anti-TNFα polyclonal antibody (5 µg/ml) was added (medium plus anti-TNFαAb). *p<0.0001. (D). Expression of CD20 on the cell surface of C1R, Raji and CLL cells (one patients out of ten analyzed) or HER2 on SKBR3 and BT474 breast adenocarcinomas cell lines upon incubation for three days with solvent (upper panels) or 10 µM of fluvastatin (lower panels). Cells were labelled with either anti-CD20 mAb or anti-HER2 mAb followed by alexafluor647 conjugated isotype specific GAM (thick lines). Thin line: cells labelled with an unrelated mAb matched for isotype. Results are expressed as Log far red fluorescence intensity (a.u.) vs number of cells and are representative of three independent experiments for CIR, Raji, SKBR3 and BT474 cell lines or 10 CLL patients. (E). ADCC mediated by ex-vivo NK cells incubated for 36 h with solvent or 10 µM fluvastatin, as indicated, of CIR or SKBR3 cell lines untreated (white columns) or treated with fluvastatin (for three day at 10 µM, black columns). Results are expressed as %^51^Cr specific release and are the mean±SD of 6 donors of NK cells.

It is still debated whether patients treated with statins are more or less prone to occurrence of neoplasia and whether statin treatment can affect the therapy outcome of these patients [15], [16]; thus, we analyzed whether fluvastatin can influence ADCC elicited through the engagement of human FcγRIIIA (namely CD16) on NK cells in two different experimental settings: a) lysis of CD20^+^ C1R lymphoma target cells with the anti-CD20 rituximab; b) killing of HER2^+^ SKBR3 breast adenocarcinoma cell line using the anti-HER2 antibody trastuzumab. In these experiments, we used CD16^+^ NK cell clones expanded with IL2 as more homogeneous effector cells than primary or short-term IL2 activated NK cells. Representative NK cell clones derived from different healthy donors (results from 30 clones from 6 donors) displayed a strong ADCC of either C1R or SKBR3 tumor cell lines using anti-CD20 or anti-HER2 antibody respectively (fig. 6A). Incubation of NK cell clones for 72 h with 10 µM of fluvastatin inhibited by 50% the ADCC triggered through anti-CD20 or anti-HER2 humanized antibodies. No effect was found using 1 µM fluvastatin.

Then, we analyzed whether the release of TNFα mediated by FcγRIIIA engagement during interaction with CIR or SKBR3 in the presence of rituximab or trastuzumab respectively, can be inhibited in NK cells cultured with fluvastatin. We found that fluvastatin did not affect the release of TNFα at any dose tested (fig. 6B) and the TNFα present in culture SN was functional (fig. 6C).

### Analysis of ADCC of Tumor Target Cells Treated with Fluvastatin

It has been reported that blocking of HMG-CoA reductase with statins can affect the expression of CD20 on lymphoma cell lines and primary lymphoma cells [41] and the consequent ADCC triggered with the humanized antibody rituximab. Thus, we further tested whether statin treatment of tumor target cells can influence the efficiency of NK cell mediated killing initiated through the engagement of FcγRIIIA. First, we analyzed whether fluvastatin could affect CD20 expression on the lymphoma tumor cell lines C1R and Raji and on primary CLL (n = 10) or HER2 expression on breast adenocarcinoma cell lines SKBR3 and BT474. Fluvastatin, at 10 µM, strongly down-regulated CD20 expression on the lymphoma cell lines analyzed but not on primary CLL (fig. 6D). On the other hand, SKBR3, but not BT474, adenocarcinomas slightly downregulated HER2 surface expression upon treatment with fluvastatin (fig. 6D). More importantly the marked downregulation of CD20 on C1R cells or the slightly decrease of HER2 on SKBR3 did not affect the degree of ex-vivo NK cell-mediated ADCC (fig. 6E). In addition, when ex-vivo NK cells were treated with 10 µM of fluvastatin for 36 h, the ADCC triggered with rituximab of CD20^+^ C1R as well as that exerted against SKBR3 in the presence of trastuzumab was not affected (fig. 6E).

## Discussion

**Figure 7:**
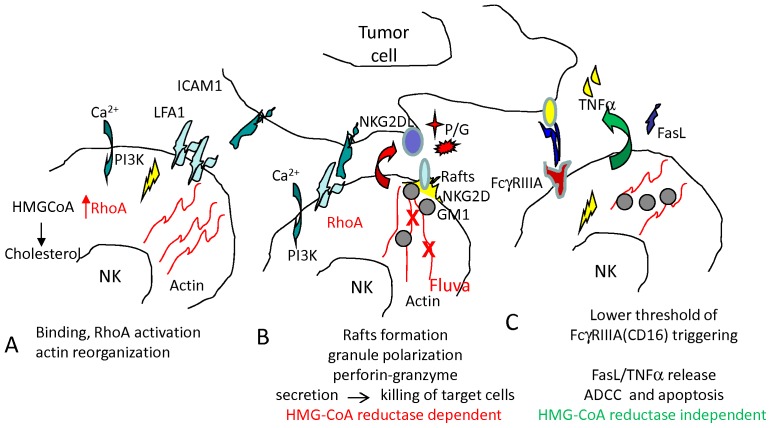
Scheme of the effects of HMG-CoA reductase inhibitors on NK cell function. Adhesion between NK and target cells triggers an activating signal in NK cells, leading to RhoA activation which favours reorganization of actin microfilaments and granule polarization to NK-target contact (A,B). At the site of contact are formed membrane microdomains (rafts) containing GM1 where activating receptor (i.e. NKG2D)-mediated [Ca^2+^]_i_ increase and activation of akt leads to the degranulation of perforin (P) and granzymes (G) and consequent lysis of target cells (B). Cholesterol is essential for membrane mobility and correct aggregation of signalling molecules in membrane rafts. Fluvastatin-mediated inhibition of HMG-CoA reductase inhibiting mevalonate synthesis causes a low cholesterol content of cell membrane, a lower mobility and aggregation of activating receptors which cannot allow granule polarization. C: Apparently, both FasL and TNFα release are conserved in fluvastatin-incubated NK cells. This may depends on their low dependency of [Ca^2+^]_i_ increase and akt activation and on low relevance of contact site–directed secretion. Indeed, when released FasL and TNFα may kill targets if these targets express the corresponding receptors. NK cell activation via FcγRIIIa (CD16), as it can occur with therapeutic antibodies rituximab or trastuzumab, has a quite low threshold of activation and only very high doses of fluvastatin are effective. Thus, ADCC is efficient also when the cholesterol content of cell membrane is reduced.

Herein, we show that the inhibition of HMG-CoA reductase with fluvastatin, leading to the block of mevalonate pathway, affects perforin/granzyme release but not FasL and TNFα secretion in human NK cells. Consequently, NK cells can still kill tumor targets through FasL/Fas interaction and/or TNFα-mediated cytotoxicity. Altogether, these findings indicate that interference with mevalonate pathway and cholesterol synthesis can affect some but not all the cellular immune mechanisms involved in the elimination of tumor cells. Fluvastatin inhibits LFA1-dependent RhoA activation, actin redistribution and formation of membrane rafts containing ganglioside M1; this may elucidate the molecular mechanism of the reported inhibiting effect on LFA1-mediated polarization of cytotoxic granules [26]. Indeed, during the recognition phase of target cells LFA1 is localized at the site of contact between NK and target cells and delivers the signal responsible for granule polarization that follows cytoskeleton reorganization; these processes are impaired in fluvastatin-cultured NK cells, so that the lethal hit to target cells is not efficiently delivered (fig. 7). It has been previously shown that only the lipophilic mevastatin, but not the hydrophylic pravastatin, can downregulate perforin-mediated cytolysis [26] and this is accompanied by down-regulation of LFA1 expression [25]; also, it has been shown that LFA1-dependent polarization of perforin containing granules is impaired in mevastatin-treated NK cells [26]. In our hands, lipophilic fluvastatin, but not hydrophilic pravastatin can efficiently reduce cholesterol membrane content in NK cells. This finding would suggest that the inefficacy of pravastatin vs mevastatin and/or fluvastatin depends on the different efficiency of these drugs in blocking HMG-CoA reductase activity. Further, we show that in NK cells fluvastatin can alter actin redistribution and activation of RhoA consequent to LFA1 engagement by its ligand ICAM1, more than the intensity of LFA1 surface expression. This indicates that blocking of mevalonate synthesis leads to the impairment of cytoskeleton assembly needed for the polarization of cytotoxic granules [26] (fig. 7). Thus, our and previous findings consistently point out the relevant role of mevalonate pathway in LFA1-dependent interaction between NK cells and tumor targets.

We also found that receptor mediated-[Ca^2+^]_i_ increase and phosphorylation of akt1/PKB consequent to PI3K activation are impaired in NK cells upon treatment with fluvastatin; this could explain the inhibition of lysis of different tumor targets in a short-time cytolytic assay. Indeed, we and others have reported that [Ca^2+^]_i_ increase and PI3K are important for the lethal hit delivered by NK cells directly to tumor target cells [8], [35]. This finding is in line with the observed reduction of activating receptor-induced formation of GM1 rafts, which indeed are microdomains of cell membrane involved in calcium signalling [39], [40] (fig. 7). Thus, one may propose that the reduction of cholesterol content in NK cell membrane can affect the mobility of surface receptors able to recruit signal transducers, leading to an impairment of early [Ca^2+^]_i_ increase consequent to receptor triggering.

In a previous report, it has been shown that activating receptor-mediated [Ca^2+^]_i_ increase was not affected after inhibition of HMG-CoA reductase. These results, apparently are in contrast with ours. These discrepancies may be dependent on the heterogeneity of primary NK cell populations obtained from different donors and stimulated in vitro with IL2 or on the method used for [Ca^2+^]_i_ evaluation. However, we found that fluvastatin can inhibit [Ca^2+^]_i_ increase using two different calcium probes (Fura2 or Fluo-4). This inhibiting effect is in line with the idea that co-engagement of activating receptors in rafts is not efficient in fluvastatin treated NK cells (fig. 7). In addition, we found that the percentage of responding cells, in which [Ca^2+^]_i_ increase was detected, diminished in fluvastatin-cultured NK cells depending on the triggering molecule: indeed, low (1 µM) concentrations of fluvastatin can significantly affect NKG2D- or DNAM1-mediated [Ca^2+^]_i_ increase while CD16 signal was affected only at high doses (10 µM). The different sensitivity to statins of the different activating receptors in NK cells was confirmed analyzing NK cell mediated cytolysis. Indeed, NKG2D- and DNAM1-, but not FcγRIIIA-triggered tumor cell lysis, are strongly inhibited also at low (1 µM) fluvastatin concentration. This may be related to a different threshold of activation among NKG2D or DNAM1 and FcγRIIIA on NK cells, as triggering through NKG2D or DNAM1 engagement can be achieved only using amounts of specific antibody 100–200 fold higher than those needed for triggering FcγRIIIA (fig. 7). This would indicate that the engagement of a few molecules of FcγRIIIA can deliver an efficient activating signal for cytotoxicity; accordingly, short-term cytolysis of tumor targets through the humanized antibodies rituximab or trastuzumab, is not inhibited at 1 µM fluvastatin concentration and only a 50% inhibition was found at 10 µM.

It has been shown that statins down-regulate the binding of anti-CD20 antibodies to CD20^+^ lymphoma cell lines or primary lymphoma cells, impairing the consequent ADCC and/or complement dependent cytotoxicity triggered with rituximab [41]. We found that fluvastatin can markedly downregulate CD20 expression on lymphoma cell lines but not on primary CLL cells. However, this downregulation was not able to diminish the ADCC of CD20^+^ tumor cells exerted by ex-vivo NK cells triggered with rituximab, even if NK cells were treated with fluvastatin. On the other hand, fluvastatin only marginally decreased the expression of HER2 on breast adenocarcinoma cell lines without affecting ADCC elicited by trastuzumab. Altogether, these findings suggest that ADCC can be still efficient when HMG-CoA reductase is inhibited in both tumor and NK cells. The discrepancies between our results and those reported previously [41] may depend on the different leukocyte populations and dose of rituximab used in ADCC assay. Indeed, in the above mentioned paper, PBMC-mediated ADCC was evaluated and the effect of statin was found when suboptimal doses of rituximab were used (0.001–0.1 µg/ml) [41], whereas we focused on NK cells, the strongest ADCC effectors [42], at rituximab doses compatible with the plasma concentrations found at the steady state of the drug (about 2.5 µg/ml) [43], [44].

The finding that FasL release and FasL/Fas mediated long-term cytotoxicity are not inhibited in NK cells treated with fluvastatin, indicates that tumor cells can be killed also when perforin/granzyme release is impaired. These results are in line with the reported finding that statins do not alter FasL transcription in NK cells [25]. It is conceivable that the different effect of fluvastatin on perforin/granzyme vs FasL release is related to the storage of perforin and FasL in different intracellular vesicles; indeed, we found that about one third of vesicles in NK cells can contain alternatively perforin or FasL.

Several reports pointed out that statins can block tumor cell proliferation and induce apoptosis but these drugs may also exert an immunosuppressive action and this is a major drawback of their employment in cancer treatment [15], [16]. Our findings would indicate that tumor cell apoptosis induced through Fas/FasL interaction can be efficient also in the presence of high statin concentrations. We are aware that the effects observed on NK cell function were detected at doses of fluvastatin (10-1 µM) not compatible with the plasma levels found during clinical management of hypercholesterolemia (10–100 nM) [45]. However, the high doses used, also by others [41], are in line with those effective in blocking cell proliferation and inducing tumor cell apoptosis [46]. Thus, our finding supporting a different sensitivity between CD16/FcγRIIIA activation and triggering mediated by activating NK cell receptors is of relevance, as in the tumor microenvironment higher concentration of a drug can be achieved using appropriate drug-delivery systems [47].

Interestingly, we found that fluvastatin did not inhibit the release of TNFα elicited by NK-tumor cell target interaction or engagement of FcγRIIIA. We should point out that fluvastatin induces an increase of TNFα release in NK-K562 co-cultures while no increment was observed using Jurkat cells as targets. This different behaviour of fluvastatin may depend on the lack of inhibiting signal (in the case of HLAI- K562 cells) delivered through HLAI which is present upon NK-Jurkat cell interaction. Further, fluvastatin did not affect NKG2D-mediated TNFα release with a dose still efficient in blocking NKG2D-mediated cytolysis. Although it is to be still elucidated the mechanism by which fluvastatin can increase or not TNFα release, our findings support the notion that this cytokine may be differently regulated when HMG-CoA reductase is inhibited. Recently, it has been shown that HMG-CoA reductase inhibitors can even stimulate NK cells to produce IFNγ [28], indicating that statins may also trigger cytokine production. This effect was referred to the presence in the peripheral blood of a CD14^+^CD56^+^ dendritic cell population which seems responsible for the triggering of IFNγ production in NK cells. However, in our experimental setting, no CD14^+^CD56^+^ dendritic cells were found among ex-vivo isolated NK cells (not shown) and such cells were also absent in our in vitro cultures where NK cell clones treated with fluvastatin could secrete TNFα upon activation.

In conclusion, our present findings indicate that interference with mevalonate synthesis pathway using fluvastatin can selectively inhibit only some molecular mechanisms responsible for killing of tumor cells and that this inhibiting effect is less evident on FcγRIIIA/CD16-mediated NK cell triggering. Thus, fluvastatin may be used in tumor treatment sparing in NK cells the activating receptor-mediated release of antitumor soluble factors as FasL and TNFα and ADCC activity.

## Supporting Information

Figure S1 **Effect of fluvastatin on NK cell mediated cytolysis and cholesterol content in NK cells.** (A). Cytolytic activity of NK cells against K562 cell line was analyzed in a 4 hr ^51^Cr release assay. Left panel: Fluvastain was added either during the cytolytic assay (4 h) or to NK cells for 36 h (36 h) or 3d or 6d together with IL2 (3d+IL2 or 6d+IL2), before the assay. Some experiments were performed by adding saturating amount of anti-LFA1 and anti-ICAM1 mAbs (5 µg/ml) at the onset of the cytolytic assay. Right panel: Cytolytic activity of NK cells cultured for 6d+IL2 with solvent or fluvastatin (10-1.0-0.1 µM), or fluvastatin and mevalonate, against the indicated cell lines. (B). Cytolysis of K562 or FO1 or U937 cell lines of NK cells treated with 10 µM of atorvastatin or mevastatin or simvastatin or pravastatin. (C). Quantification of membrane cholesterol present in NK cells cultured in solvent of fluvastatin (DMSO, 1∶1000 in culture medium) or with fluvastatin (10-1-0.1 µM) and in solvent of pravastatin (H_2_O, diluted 1∶1000 in culture medium) or with pravastatin at the same concentrations. Results are expressed as µg/10^6^cells.
(TIF)

Figure S2 **Fluvastatin effects on NK cell-mediated cytolysis triggered through activating receptors.** Cytolysis of ex-vivo isolated NK cells (A) or NK cells cultured for 6d+IL2 was assessed in a redirected killing assay with the P815 target cell line. Either ex-vivo NK cells or IL2-cultured NK cells were incubated for 36 h or cultured for 6d with the indicated drugs or solvent (DMSO). Then, cytolysis of P815 cells was triggered with mAbs to the indicated receptors and analyzed in a 4 h ^51^Cr release assay at the E:T ratio of 10∶1 (A) or 1∶1 (B). UnmAb: unrelated mAb matched for isotype as negative control. Basal: cytolysis detected in the absence of any mAb. Results are expressed as percentage of ^51^Cr specific release and are the mean±SD of six experiments.
(TIF)

Figure S3 **Effect of fluvastatin on NK cell surface markers expression.** NK cells isolated from peripheral blood (n = 6) were cultured in medium alone (A, left dot plots and B) or supplemented with IL2 (10 ng/ml) (A, right dot plots and C), with solvent of fluvastatin (solvent, DMSO 1∶1000 diluted) or fluvastatin (0.1-1-10 µM) for 3d. (A). Forward and side scatter analysis of NK cells, R1: gate on living cells. (B and C). Surface expression of the indicated molecules (black thick line) on R1 gated NK cells evaluated by indirect immunofluorescence using the specific mAbs followed by PE-GAM. NK cells stained with an unrelated mAb as negative control are indicated by the black thin line histogram. Samples were run on a CyAnADP flow cytometer and results are expressed as Log red fluorescence intensity (MFI, in arbitrary units: a.u.) vs number of cells. In each subpanel MFI of cells stained with the corresponding mAb is indicated. (D,E). NK cells cultured with IL2 in medium alone (medium) or as in panel C were analyzed on day 6 for the indicated activating (CD16, NKG2D and DNAM1, D) or inhibiting (KIR2D, CD94 and LAIR1, E) cell surface receptors with specific mAbs. Samples were run on a CyAnADP flow cytometer. Results are expressed as mean Log red fluorescence intensity (MFI, a.u.) and are the mean±SD from 6 independent experiments. Statistical significance ***p<0.0001 **p<0.001 versus control. ns: not significant.
(TIF)

Figure S4 **CD107a, perforin, FasL localization in NK cells.** (A) IL2-cultured NK cells were cyto-centrifuged, fixed, permeabilized and stained with anti-perforin and anti-calnexin (as a marker for endoplasmic reticulum) or anti-FasL or anti-CD107a mAb followed by isotype specific GAM conjugated with alexafluor488 (perforin) or with alexafluor647 (calnexin or FasL or CD107a) and analyzed by confocal microscopy. (B). IL2-cultured NK cells were triggered with anti-NKG2D and GAM for 15 min, cyto-centrifuged, fixed, permeabilized and stained with specific mAbs to the indicated molecules (Perforin green, FasL red) and analyzed by confocal microscopy (Olympus FV500). Neg control: NK cells without mAbs. Images were taken with FluoView computer program using 40X/1.40NA planapo oil objective. 400X magnifiication. (C and D): 3x zoom of white squares in panel B. White Bar: 10 µm. Arrows indicate granules containing either FasL or Perforin (C), or both (D). (E). Analysis of FasL^+^ or perforin^+^ or FasL-perforin double positive granules evaluated in at least 40 NK cells from three different donors. Counting of granules was performed using analysis SYS program upon microscopic observation. Images were taken with Cell^R^ (Olympus) imagine analysis system using 40X/1.40NA planapo oil objective.
(TIF)

File S1 In this file, we describe the effect of fluvastatin on a) NK-cell mediated cytolysis of tumor cells; b) NK-cell mediated cytolysis triggered through specific receptors; and c) the surface expression of receptors involved in NK-tumor target cell interaction and triggering of cytolysis.
(DOC)
