# Supplementary figures and images for: Selective Role of Mevalonate Pathway in Regulating Perforin but Not FasL and TNFalpha Release in Human Natural Killer Cells
Source: PLoS One. 2013 May 7;8(5):e62932. doi: 10.1371/journal.pone.0062932 (PMC3646988; doi:10.1371/journal.pone.0062932)

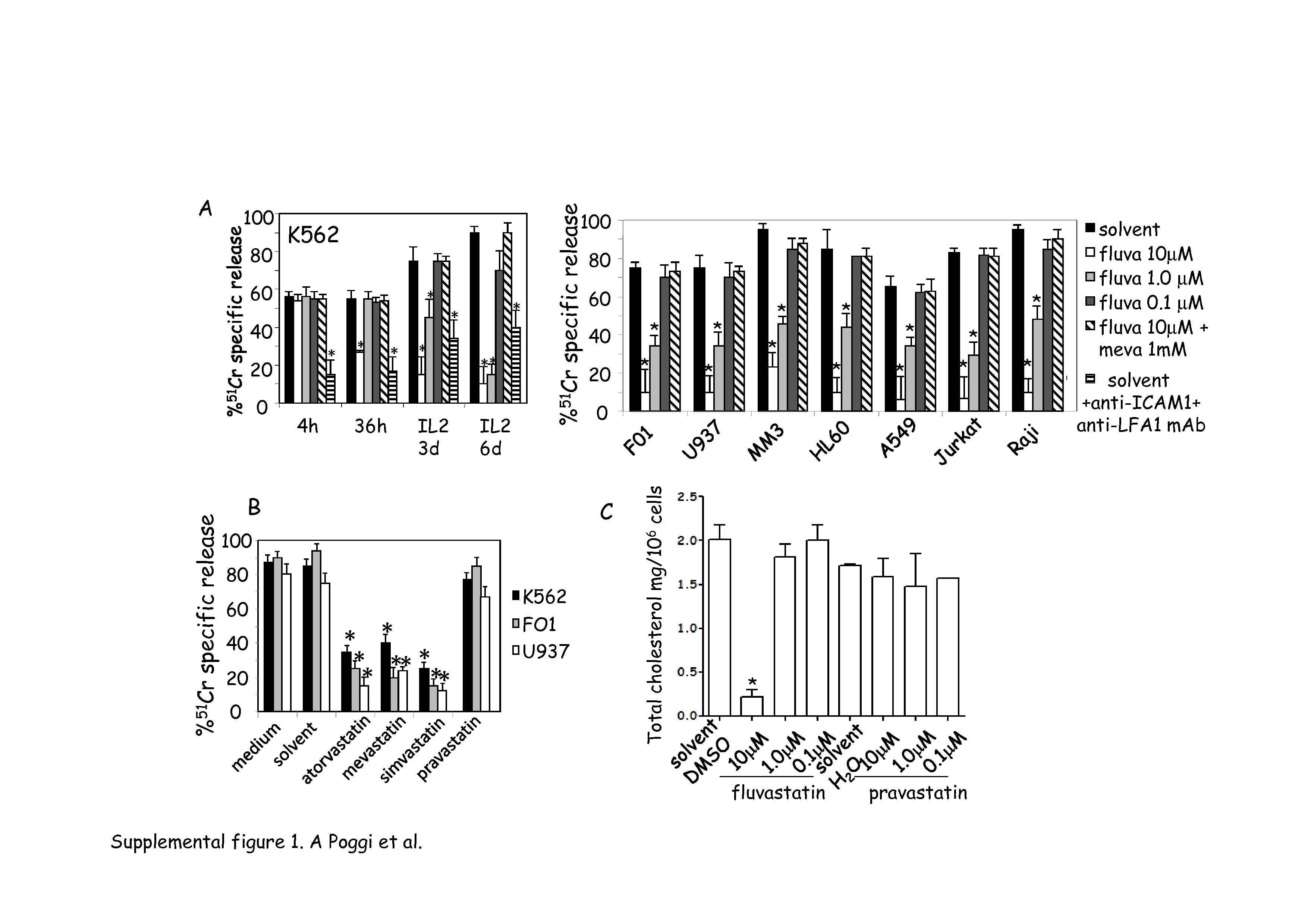

Supplement: Figure S1 — Effect of fluvastatin on NK cell mediated cytolysis and cholesterol content in NK cells. (A). Cytolytic activity of NK cells against K562 cell line was analyzed in a 4 hr 51Cr release assay. Left panel: Fluvastain was added either during the cytolytic assay (4 h) or to NK cells for 36 h (36 h) or 3d or 6d together with IL2 (3d+IL2 or 6d+IL2), before the assay. Some experiments were performed by adding saturating amount of anti-LFA1 and anti-ICAM1 mAbs (5 µg/ml) at the onset of the cytolytic assay. Right panel: Cytolytic activity of NK cells cultured for 6d+IL2 with solvent or fluvastatin (10-1.0-0.1 µM), or fluvastatin and mevalonate, against the indicated cell lines. (B). Cytolysis of K562 or FO1 or U937 cell lines of NK cells treated with 10 µM of atorvastatin or mevastatin or simvastatin or pravastatin. (C). Quantification of membrane cholesterol present in NK cells cultured in solvent of fluvastatin (DMSO, 1∶1000 in culture medium) or with fluvastatin (10-1-0.1 µM) and in solvent of pravastatin (H2O, diluted 1∶1000 in culture medium) or with pravastatin at the same concentrations. Results are expressed as µg/106cells. (TIF) [file pone.0062932.s001.tif]

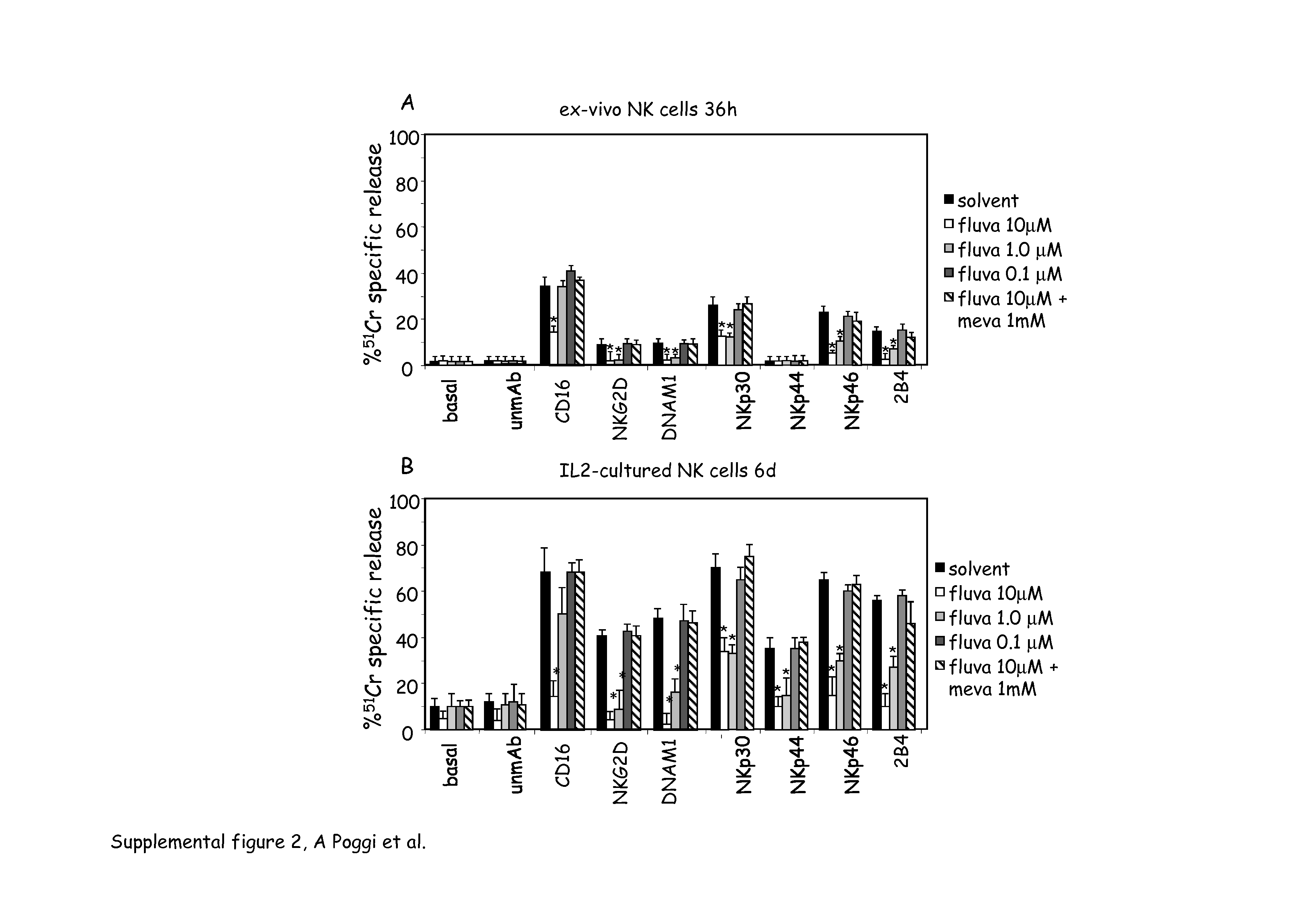

Supplement: Figure S2 — Fluvastatin effects on NK cell-mediated cytolysis triggered through activating receptors. Cytolysis of ex-vivo isolated NK cells (A) or NK cells cultured for 6d+IL2 was assessed in a redirected killing assay with the P815 target cell line. Either ex-vivo NK cells or IL2-cultured NK cells were incubated for 36 h or cultured for 6d with the indicated drugs or solvent (DMSO). Then, cytolysis of P815 cells was triggered with mAbs to the indicated receptors and analyzed in a 4 h 51Cr release assay at the E:T ratio of 10∶1 (A) or 1∶1 (B). UnmAb: unrelated mAb matched for isotype as negative control. Basal: cytolysis detected in the absence of any mAb. Results are expressed as percentage of 51Cr specific release and are the mean±SD of six experiments. (TIF) [file pone.0062932.s002.tif]

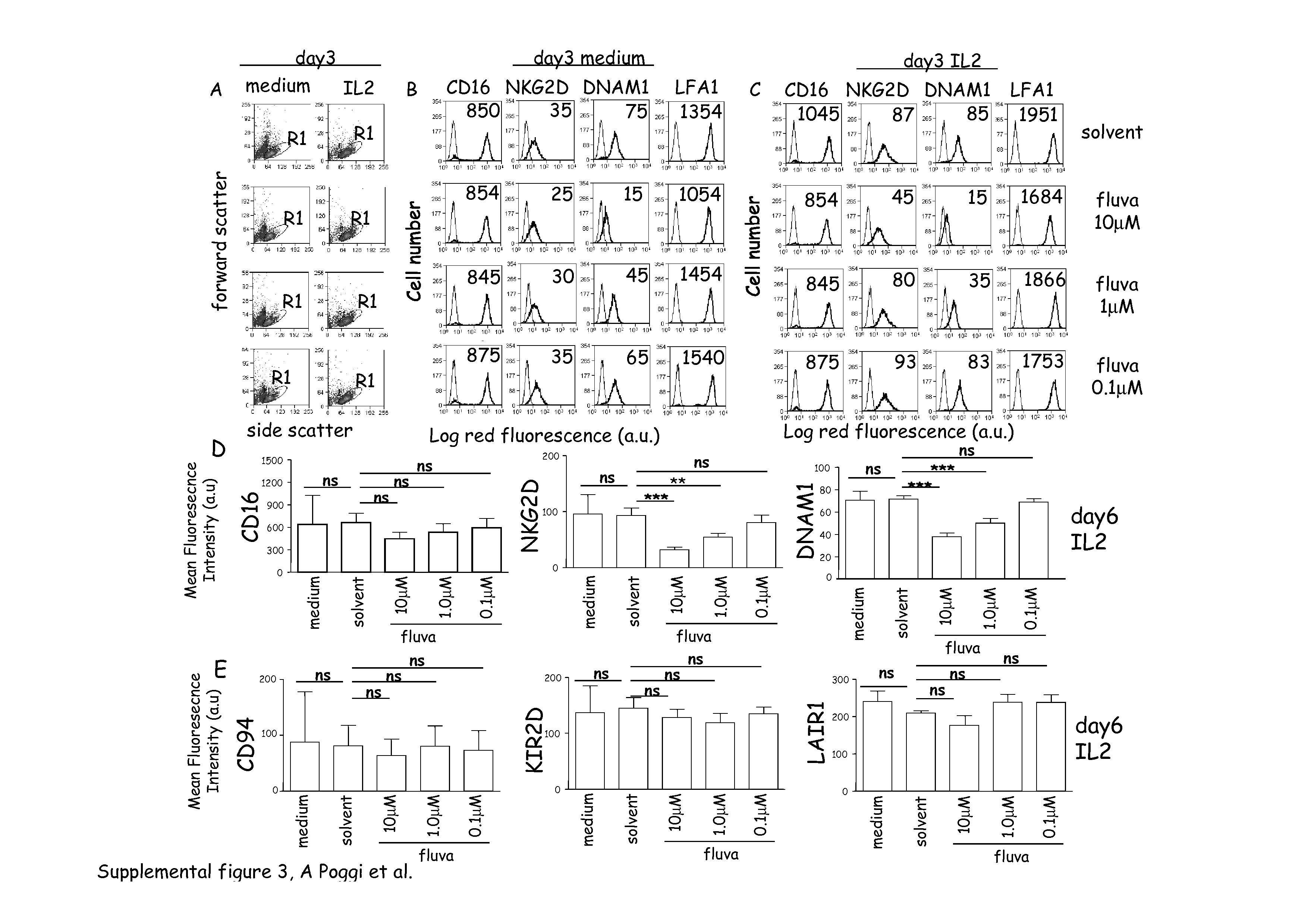

Supplement: Figure S3 — Effect of fluvastatin on NK cell surface markers expression. NK cells isolated from peripheral blood (n = 6) were cultured in medium alone (A, left dot plots and B) or supplemented with IL2 (10 ng/ml) (A, right dot plots and C), with solvent of fluvastatin (solvent, DMSO 1∶1000 diluted) or fluvastatin (0.1-1-10 µM) for 3d. (A). Forward and side scatter analysis of NK cells, R1: gate on living cells. (B and C). Surface expression of the indicated molecules (black thick line) on R1 gated NK cells evaluated by indirect immunofluorescence using the specific mAbs followed by PE-GAM. NK cells stained with an unrelated mAb as negative control are indicated by the black thin line histogram. Samples were run on a CyAnADP flow cytometer and results are expressed as Log red fluorescence intensity (MFI, in arbitrary units: a.u.) vs number of cells. In each subpanel MFI of cells stained with the corresponding mAb is indicated. (D,E). NK cells cultured with IL2 in medium alone (medium) or as in panel C were analyzed on day 6 for the indicated activating (CD16, NKG2D and DNAM1, D) or inhibiting (KIR2D, CD94 and LAIR1, E) cell surface receptors with specific mAbs. Samples were run on a CyAnADP flow cytometer. Results are expressed as mean Log red fluorescence intensity (MFI, a.u.) and are the mean±SD from 6 independent experiments. Statistical significance ***p<0.0001 **p<0.001 versus control. ns: not significant. (TIF) [file pone.0062932.s003.tif]

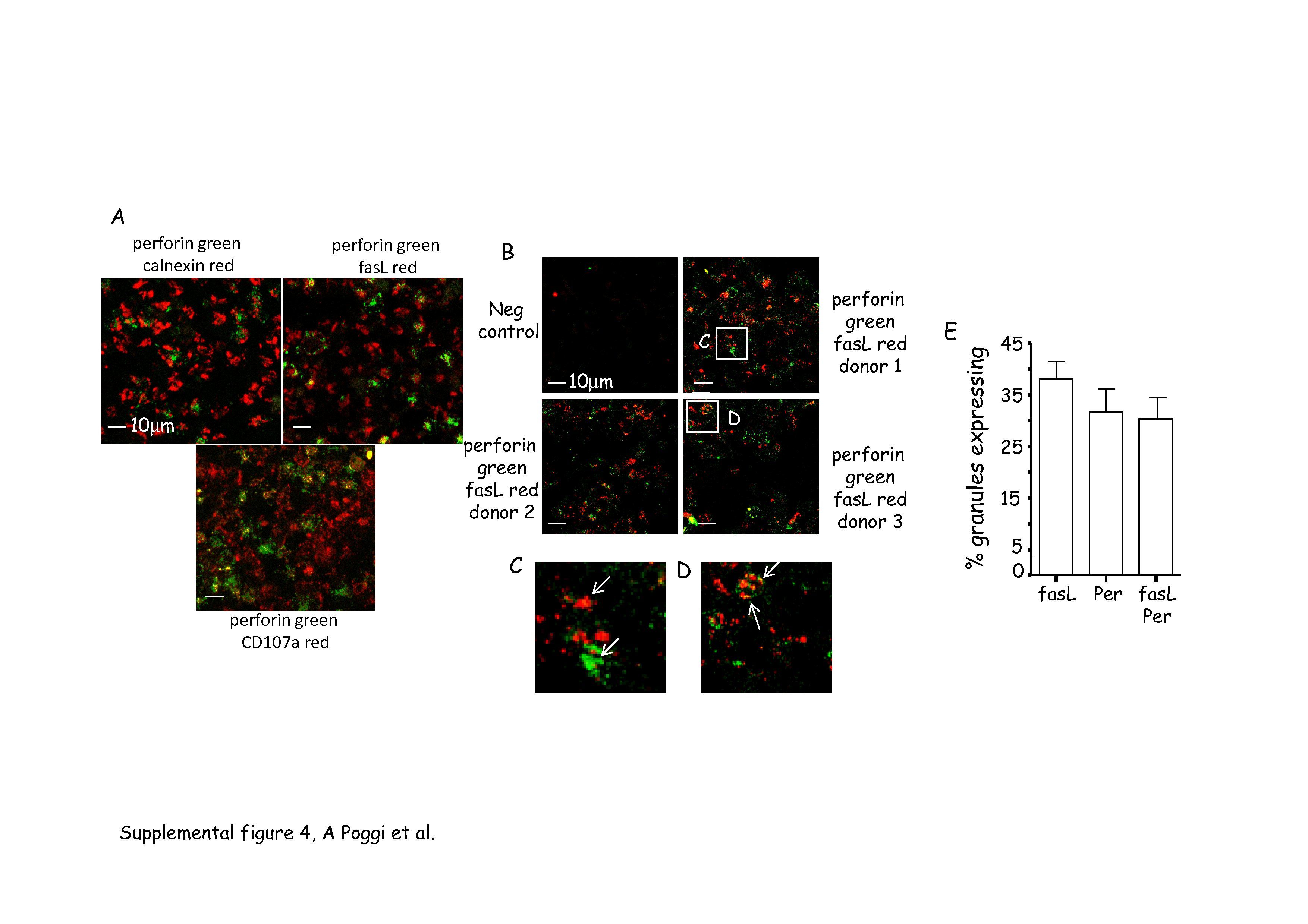

Supplement: Figure S4 — CD107a, perforin, FasL localization in NK cells. (A) IL2-cultured NK cells were cyto-centrifuged, fixed, permeabilized and stained with anti-perforin and anti-calnexin (as a marker for endoplasmic reticulum) or anti-FasL or anti-CD107a mAb followed by isotype specific GAM conjugated with alexafluor488 (perforin) or with alexafluor647 (calnexin or FasL or CD107a) and analyzed by confocal microscopy. (B). IL2-cultured NK cells were triggered with anti-NKG2D and GAM for 15 min, cyto-centrifuged, fixed, permeabilized and stained with specific mAbs to the indicated molecules (Perforin green, FasL red) and analyzed by confocal microscopy (Olympus FV500). Neg control: NK cells without mAbs. Images were taken with FluoView computer program using 40X/1.40NA planapo oil objective. 400X magnifiication. (C and D): 3x zoom of white squares in panel B. White Bar: 10 µm. Arrows indicate granules containing either FasL or Perforin (C), or both (D). (E). Analysis of FasL+ or perforin+ or FasL-perforin double positive granules evaluated in at least 40 NK cells from three different donors. Counting of granules was performed using analysis SYS program upon microscopic observation. Images were taken with CellR (Olympus) imagine analysis system using 40X/1.40NA planapo oil objective. (TIF) [file pone.0062932.s004.tif]
