# Supplementary material for: Selective Role of Mevalonate Pathway in Regulating Perforin but Not FasL and TNFalpha Release in Human Natural Killer Cells
Source: PLoS One. 2013 May 7;8(5):e62932. doi: 10.1371/journal.pone.0062932 (PMC3646988; doi:10.1371/journal.pone.0062932)
Supplement: File S1 — In this file, we describe the effect of fluvastatin on a) NK-cell mediated cytolysis of tumor cells; b) NK-cell mediated cytolysis triggered through specific receptors; and c) the surface expression of receptors involved in NK-tumor target cell interaction and triggering of cytolysis. (DOC) [file pone.0062932.s005.doc]

# Supplemental Results

***Effect of fluvastatin on NK-cell mediated cytolysis of tumor cells.***

NK cells isolated from peripheral blood can kill NK-sensitive K562 erythroleukemic cells; this killing is mostly due to the interaction of LFA1 on NK cells with ICAM1 on K562 cells respectively (Ref. 1 and Suppl.fig.1A). Ex-vivo isolated NK cells, incubated with fluvastatin during the 4h cytolytic assay exerted a cytolytic activity similar to untreated NK cells (Suppl. fig.1A, left panel). An evident inhibiting effect (range 50-75%, n=8) of NK cell-mediated cytolysis was detected after 36h of fluvastatin incubation at 10μM concentration (Suppl. fig.1A left). To evaluate the effect of statins on cytokine-activated NK cell killing, primary NK cells were incubated in culture medium with IL2, in the presence of increasing doses of fluvastatin, for 3d and 6d and cytolysis to K562 target cells was analyzed. Three day-IL2-activated NK cells efficiently killed K562 cells; this killing was strongly impaired (by 80%) in NK cells cultured with 10μM fluvastatin; on day 6, this inhibiting effect was stronger and also evident at 1μM fluvastatin (Suppl.fig.1A, left panel). Fluvastatin-dependent inhibition of cytolysis was evident using several other tumor targets, as the melanoma FO1, the lung adenocarcinoma A549, the myeloid leukemias U937, MM3 and HL60, the lymphomas Jurkat and Raji (Suppl.fig.1A, right panel). Similar results were obtained using other statins, as atorvastatin, simvastatin or mevastatin (Suppl.fig.1B). On the other hand, pravastatin did not affect NK cell-mediated cytotoxicity (Suppl.fig.1B); it has to be noted that pravastatin, different from fluvastatin, did not reduce the membrane cholesterol content of NK cells (Suppl.fig.1C).

***Effect of fluvastatin on NK-cell mediated cytolysis triggered through specific receptors.***

Next, to analyze the effect of fluvastatin also on the killing mediated through the specific activation of NK cell triggering receptors, ex-vivo isolated NK cells and IL2-cultured NK cells were assayed in redirected killing performed using mAbs directed to triggering receptors and the murine FcγR^+^ mastocytoma cell line P815. In this experimental setting, the relevance of LFA1 in E-T interaction is negligible as the bridge between the NK and target cells is artificially obtained by the mAb, mimicking the interaction between the activating receptor and its ligand (2, 29,30). Primary NK cells efficiently killed P815 target cells upon engagement of CD16 with a specific mAb and, to a lesser extent, with mAsb to either NKG2D or DNAM1 or NKp30 or NKp46 or 2B4, while triggering through NKp44 was almost undetectable (Suppl.fig.2A). Incubation of cells with 10μM of fluvastatin led to a reduction of CD16-mediated killing (range 30-40%), while killing triggered through NKG2D, DNAM1, NKp30, NKp46 and 2B4 was almost completely abolished (range 80-100%). IL-2-stimulated NK cells efficiently killed P815 target cells through the engagement of all the activating receptors analyzed, including CD16, NKG2D, DNAM1, NKp30, NKp44, NKp46 and 2B4 (Suppl.fig.2B). Re-directed killing triggered through NKG2D was markedly inhibited in NK cells cultured with 10μM or 1μM fluvastatin (95% and 90% inhibition respectively); this inhibition was not detectable at 0.1μM concentration (Suppl.fig.2B). A similar behaviour was observed when triggering was mediated through DNAM1, NKp30, NKp44, NKp46 and 2B4 (Suppl.fig.2B). CD16-mediated triggering was inhibited at 10μM fluvastatin (80-90% inhibition) but less at 1.0μM concentration (20-30% inhibition) (Suppl.fig.2B). The inhibiting effect on cytolysis was abolished when mevalonate was added to the culture together with fluvastatin (Suppl.fig.2A and B).

***Influence of fluvastatin on the surface expression of receptors involved in NK-tumor target cell interaction and triggering of cytolysis.***

We further analyzed whether fluvastatin affected the surface expression of CD16, NKG2D, DNAM1 and LFA1. NK cells cultured with medium alone for 3d showed physical parameters (FSC and SSC) similar to NK cells cultured with fluvastatin (Suppl.fig.3A) and the expression of CD16 and LFA1 was not affected (Suppl.fig.3B). The expression of NKG2D and DNAM1 was strongly reduced in fluvastatin treated cells, both when NK cells were cultured in medium alone or with IL2 (Suppl.fig.3B,C) in a dose (Suppl.fig.3D) and time (not shown) dependent manner. Of note, the IL2-dependent upregulation of NKG2D expression was markedly reduced and a similar behaviour was detected for 2B4 and NKp30 (not shown). On the other hand, the expression of receptors such as KIR2D, CD94 and LAIR1 was not altered upon culture with IL2 and fluvastatin (Suppl.fig.3E).

**Legends to Supplemental Figures**

***Supplemental figure 1. Effect of fluvastatin on NK cell mediated cytolysis and cholesterol content in NK cells.***

(A). Cytolytic activity of NK cells against K562 cell line was analyzed in a 4hr ^51^Cr release assay. Left panel: Fluvastain was added either during the cytolytic assay (4h) or to NK cells for 36h (36h) or 3d or 6d toghether with IL2 (3d+IL2 or 6d+IL2), before the assay. Some experiments were performed by adding saturating amount of anti-LFA1 and anti-ICAM1 mAbs (5μg/ml) at the onset of the cytolytic assay. Right panel: Cytolytic activity of NK cells cultured for 6d+IL2 with solvent or fluvastatin (10-1.0-0.1μM), or fluvastatin and mevalonate, against the indicated cell lines.

(B). Cytolysis of K562 or FO1 or U937 cell lines of NK cells treated with 10μM of atorvastatin or mevastatin or simvastatin or pravastatin. (C). Quantification of membrane cholesterol present in NK cells cultured in solvent of fluvastatin (DMSO, 1:1000 in culture medium) or with fluvastatin (10-1-0.1μM) and in solvent of pravastatin (H_2_O, diluted 1:1000 in culture medium) or with pravastatin at the same concentrations. Results are expressed as μg/10^6^cells.

***Supplemental figure 2. Fluvastatin effects on NK cell-mediated cytolysis triggered through activating receptors.***

Cytolysis of ex-vivo isolated NK cells (A) or NK cells cultured for 6d+IL2 was assessed in a redirected killing assay with the P815 target cell line. Either ex-vivo NK cells or IL2-cultured NK cells were incubated for 36h or cultured for 6d with the indicated drugs or solvent (DMSO). Then, cytolysis of P815 cells was triggered with mAbs to the indicated receptors and analyzed in a 4h ^51^Cr release assay at the E:T ratio of 10:1 (A) or 1:1 (B). UnmAb: unrelated mAb matched for isotype as negative control. Basal: cytolysis detected in the absence of any mAb. Results are expressed as percentage of ^51^Cr specific release and are the mean±SD of six experiments.

***Supplemental figure 3. Effect of fluvastatin on NK cell surface markers expression.***

NK cells isolated from peripheral blood (n=6) were cultured in medium alone (A, left dot plots and B) or supplemented with IL2 (10ng/ml) (A, right dot plots and C), with solvent of fluvastatin (solvent, DMSO 1:1000 diluited) or fluvastatin (0.1-1-10μM) for 3d. (A). Forward and side scatter analysis of NK cells, R1: gate on living cells. (B and C). Surface expression of the indicated molecules (black thick line) on R1 gated NK cells evaluated by indirect immunofluorescence using the specific mAbs followed by PE-GAM. NK cells stained with an unrelated mAb as negative control are indicated by the black thin line histogram. Samples were run on a CyAnADP flow cytometer and results are expressed as Log red fluorescence intensity (MFI, in arbitrary units: a.u.) vs number of cells. In each subpanel MFI of cells stained with the corresponding mAb is indicated. (D,E). NK cells cultured with IL2 in medium alone (medium) or as in panel C were analyzed on day 6 for the indicated activating (CD16, NKG2D and DNAM1, D) or inhibiting (KIR2D, CD94 and LAIR1, E) cell surface receptors with specific mAbs. Samples were run on a CyAnADP flow cytometer. Results are expressed as mean Log red fluorescence intensity (MFI, a.u.) and are the mean±SD from 6 independent experiments. Statistical significance ***p<0.0001 **p<0.001 versus control. ns: not significant.

***Supplemental figure 4. CD107a, perforin, FasL localization in NK cells.***

(A) IL2-cultured NK cells were cyto-centrifuged, fixed, permeabilized and stained with anti-perforin and anti-calnexin (as a marker for endoplasmic reticulum) or anti-FasL or anti-CD107a mAb followed by isotype specific GAM conjugated with alexafluor488 (perforin) or with alexafluor647 (calnexin or FasL or CD107a) and analyzed by confocal microscopy. (B). IL2-cultured NK cells were triggered with anti-NKG2D and GAM for 15min, cyto-centrifuged, fixed, permeabilized and stained with specific mAbs to the indicated molecules (Perforin green, FasL red) and analyzed by confocal microscopy (Olympus FV500). Neg control: NK cells without mAbs. Images were taken with FluoView computer program using 40X/1.40NA planapo oil objective. 400X magnifiication. (C and D): 3x zoom of white squares in panel B. White Bar: 10μm. Arrows indicate granules containing either FasL or Perforin (C), or both (D). (E). Analysis of FasL^+^ or perforin^+^ or FasL-perforin double positive granules evaluated in at least 40 NK cells from three different donors. Counting of granules was performed using analysis SYS program upon microscopic observation. Images were taken with Cell^R^ (Olympus) imagine analysis system using 40X/1.40NA planapo oil objective.
